# Interaction-induced particle-hole symmetry breaking and fractional exclusion statistics

**DOI:** 10.1093/nsr/nwac027

**Published:** 2022-02-24

**Authors:** Xibo Zhang, Yang-Yang Chen, Longxiang Liu, Youjin Deng, Xiwen Guan

**Affiliations:** International Center for Quantum Materials, School of Physics, Peking University, Beijing 100871, China; Collaborative Innovation Center of Quantum Matter, Beijing 100871, China; Beijing Academy of Quantum Information Sciences, Beijing 100193, China; State Key Laboratory of Magnetic Resonance and Atomic and Molecular Physics, Wuhan Institute of Physics and Mathematics, Innovation Academy for Precision Measurement Science and Technology, Chinese Academy of Sciences, Wuhan 430071, China; Institute of Modern Physics, Northwest University, Xi’an 710127, China; Chinese Academy of Sciences Center for Excellence in Quantum Information and Quantum Physics, University of Science and Technology of China, Hefei 230326, China; Chinese Academy of Sciences Center for Excellence in Quantum Information and Quantum Physics, University of Science and Technology of China, Hefei 230326, China; MinJiang Collaborative Center for Theoretical Physics, College of Physics and Electronic Information Engineering, Minjiang University, Fuzhou 350108, China; State Key Laboratory of Magnetic Resonance and Atomic and Molecular Physics, Wuhan Institute of Physics and Mathematics, Innovation Academy for Precision Measurement Science and Technology, Chinese Academy of Sciences, Wuhan 430071, China; Department of Theoretical Physics, Research School of Physics and Engineering, Australian National University, Canberra ACT 0200, Australia

**Keywords:** quantum statistics, interaction, particle-hole symmetry breaking, fractional exclusion statistics, strongly correlated quantum materials

## Abstract

Quantum statistics plays a fundamental role in the laws of nature. Haldane fractional exclusion statistics (FES) generalizes the Pauli exclusion statistics, and can emerge in the properties of elementary particles and hole excitations of a quantum system consisting of conventional bosons or fermions. FES has a long history of intensive studies, but its simple realization in interacting physical systems is rare. Here we report a simple non-mutual FES that depicts the particle-hole symmetry breaking in interacting Bose gases at a quantum critical point. We show that the FES distribution directly comes from particle-hole symmetry breaking. Based on exact solutions, quantum Monte Carlo simulations and experiments, we find that, over a wide range of interaction strengths, the macroscopic physical properties of these gases are determined by non-interacting quasi-particles that obey non-mutual FES of the same form in one and two dimensions. Whereas strongly interacting Bose gases reach full fermionization in one dimension, they exhibit incomplete fermionization in two dimensions. Our results provide a generic connection between interaction-induced particle-hole symmetry breaking (depicted by FES) and macroscopic properties of many-body systems in arbitrary dimensions. Our work lays the groundwork for using FES to explore quantum criticality and other novel many-body phenomena in strongly correlated quantum systems.

## Teaser

Near a quantum critical point, the macroscopic properties of interacting many-body Bose gases in one and two dimensions are determined by ideal particles that obey non-mutual fractional exclusion statistics.

## INTRODUCTION

Bose-Einstein and Fermi-Dirac statistics constitute two cornerstones of quantum statistical mechanics. However, they are not the only possible forms of quantum statistics [1]. In two dimensions, anyonic excitations can carry fractional charges and obey fractional statistics [2–7]. To generalize fractional statistics, Haldane formulated a theory of fractional exclusion statistics (FES) that continuously interpolates between Bose and Fermi statistics in arbitrary spatial dimensions [8]. This theory depicts how much the Hilbert space dimensionality for available single-particle states, namely the ‘number of holes’ (*N*_h, α_) of species α decreases as particles of species β are added to a system [8–10]:

(1) \begin{eqnarray*} \Delta N_{\mathrm{h},\alpha } & = & -\sum _{\beta } g_{\alpha \beta } \Delta N_{\mathrm{P},\beta }. \end{eqnarray*}

Here the FES parameter *g*_αβ_ is independent of the particle number *N*_P, β_. Bose and Fermi statistics correspond to the non-mutual FES where *g*_αβ_ = *g*δ_αβ_ with *g* = 0 and 1, respectively. FES has found exact realizations in a few one-dimensional (1D) systems, including the Calogero-Sutherland model of particles interacting through a 1/*r*^2^ potential [11–14], Lieb-Liniger Bose gases [14,15] and anyonic gases with delta-function interaction [16,17].

FES reveals the statistical nature of a system with respect to its energy spectrum regardless of whether the constituent particles interact or not. On the other hand, particle-hole symmetry breaking (PHSB) [18] emerges as a key mechanism for understanding strongly correlated quantum materials including high-*T*_c_ superconductors [19,20] and fractional quantum Hall systems [21]. This symmetry breaking significantly influences physical properties such as equations of state [22], optical properties [23], dynamical evolutions [24], transport properties [25] and non-Fermi-liquid behaviors [26]. However, it remains challenging to identify emergent FES for depicting the particle-hole symmetry breaking in generic interacting many-body systems.

**Figure 1.**
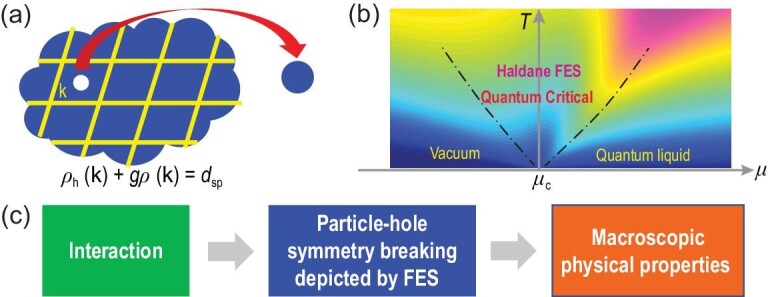
Particle-hole symmetry breaking and Haldane FES in interacting many-body systems. (a) Particle-hole symmetry breaking in a quasi-momentum cell at $\mathbf {k}$ (see Equation (2)) in an interacting system. (b) A many-body system near a quantum critical point. (c) Schematic of the logic flow: interaction determines the particle-hole symmetry breaking (depicted by FES) that in turn governs the macroscopic physical properties of a many-body system at and near a quantum critical point.

In this article, we show that FES naturally emerges as a result of particle-hole symmetry breaking in quantum many-body systems (see Fig. 1(a) and Equation (2)). In particular, we demonstrate interaction-induced non-mutual FES at a quantum critical point. We consider a repulsively interacting Bose gas that undergoes a quantum phase transition under zero temperature (*T* = 0) from a vacuum to a quantum liquid when the chemical potential μ exceeds a critical value μ_c_ (Fig. 1(b)). Here ‘quantum liquid’ denotes a Tomonaga-Luttinger liquid (TLL) [27] in one dimension or a superfluid in higher dimensions [28]. Based on exact solutions in one dimension and high-precision quantum Monte Carlo (QMC) simulations in two dimensions, we report evidence for emergent particle-hole symmetry breaking (Equation (2)) in these many-body systems. Our results are further supported by existing experimental data given in [27–32]. We establish a one-to-one correspondence between interaction and particle-hole symmetry breaking over a wide range of interaction strengths, and further observe that, remarkably, such symmetry breaking determines the macroscopic properties of interacting gases in a unified manner, as summarized by the logic flow shown in Fig. 1(c).

### Particle-hole symmetry breaking and FES

In a quantum many-body system, interaction dresses the constituent particles to form quasi-particles that are statistically distributed over the quasi-momentum space. In each quasi-momentum cell that defines the species in Equation (1), the number of unoccupied states depends on the numbers of occupied states in the same cell and in other cells, forming a net of correlated cells in general. Such correlated cells can be depicted by quasi-momentum-dependent particle-hole symmetry breaking; see Equation S43 within the online supplementary material. At a quantum critical point where the system is strongly correlated with large characteristic lengths in real space, these quasi-momentum cells become decoupled into nearly independent cells (see Section 3 within the online supplementary material), which leads to a simplified form of particle-hole symmetry breaking equation that we expect to hold in arbitrary dimensions (Fig. 1(a)):

(2) \begin{equation*} \rho _{\mathrm{h}} ({\bf k}) + g \rho ({\bf k}) =d_{\mathrm{sp}}. \end{equation*}

Here, the species label α is given by quasi-momentum $\mathbf {k}$, *g*_αβ_ by $g\delta (\mathbf {k} - \mathbf {k}^{\prime })$, *N*_P_ and *N*_h_ are scaled into distribution functions, $\rho (\mathbf {k})$ and $\rho _{\mathrm{h}}(\mathbf {k})$, of occupied states and of holes, and *d*_sp_ = 1/(2π)^*D*^ is a bare dimensionality of states in a phase-space unit cell for a *D*-dimensional system.

Accordingly, a non-mutual FES distribution [9,14] of quasi-particles naturally emerges at a quantum critical point. We prove (for details, see Section 4 within the online supplementary material) that Equation (2) directly gives rise to a non-mutual FES distribution with the following occupation number *f* in a state with energy ε:

(3) \begin{eqnarray*} f(\epsilon ) & =& \frac{1}{w(\zeta ) + g}, \\ w^g(1+w)^{1-g} & =& \zeta \equiv \exp \left( \frac{\epsilon - \mu }{T} \right). \end{eqnarray*}

This generic connection between Equations (2) and (3) enables understanding interacting systems from the perspective of emergent FES phenomena. Based on the interaction-induced particle-hole symmetry breaking (Equation (2)), the macroscopic physical properties of many-body systems can be obtained through non-interacting quasi-particles that obey the non-mutual FES distribution in Equation (3). Specifically, the number density and energy density are given by *n* = ∫*G*(ε)*f*(ε)dε and *e* = ∫*G*(ε)*f*(ε)εdε, where the density of states per volume is given by $G(\epsilon ) = {1}/{(2\pi \sqrt{\epsilon })}$ in one dimension and 1/(4π) in two dimensions for non-relativistic particles. We set 2*m* = *k*_B_ = ℏ = 1, where *m* is the particle mass, *k*_B_ the Boltzmann constant and ℏ the reduced Planck constant.

To connect Equation (2) to physical interacting systems, we introduce an interaction-FES correspondence hypothesis:

(4) \begin{eqnarray*} R_{ \mathcal {H}\mathrm{Dim}} \equiv \frac{g(\tilde{c}) - g_{ 0}}{g_{ \infty } - g(\tilde{c})} = \frac{\tilde{c}}{\tilde{c}_1}. \end{eqnarray*}

**Figure 2.**
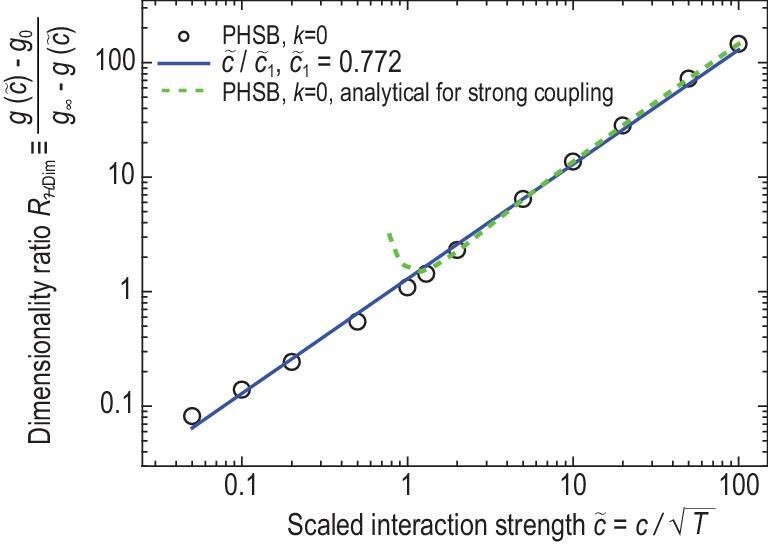
Demonstration of the proportionality relation in the interaction-FES correspondence hypothesis (Equation (4)). Based on exact solutions for 1D Bose gases with delta-function interaction, we compute $g_{{\mathrm{PHSB}, \mathbf {k}=0}}$ that depicts the particle-hole symmetry breaking in low-energy excitations. The numerical data (circles) show the proportionality relation with fitted $\tilde{c}_{{1,\mathrm{PHSB}}} = 0.77(3)$. The solid line shows Equation (4) with *c*_1_ = 0.772 (see Equation (10) for details). Under strong couplings ($\tilde{c}/\tilde{n} \gg 1$), the numerical data can be described by an analytical form (dashed line; see Equation (7)).

Here $\tilde{c}$ is a properly scaled interaction strength, $g(\tilde{c})$ is the corresponding FES parameter in Equations (2) and (3), $g_{ 0/\infty } = g(\tilde{c} = 0/\infty )$ and $\tilde{c}_1$ is a coefficient. This hypothesis is inspired by an analytical result for the FES in 1D strongly interacting Bose gases [33] as well as a Ginzburg-Landau theory for 2D superfluids [31,34], and is found to apply over a large interaction range (see Fig. 2). It provides a simple proportionality relation between the interaction strength $\tilde{c}$ and the resulting Hilbert space dimensionality ratio ($R_{ \mathcal {H}\mathrm{Dim}}$) of *g* − *g*_0_ (for the Hilbert space occupied by one single particle because of interaction) to *g*_∞_ − *g* (for the ‘remaining’ Hilbert space that is occupiable but yet unoccupied because $\tilde{c}$ has not reached infinity). For interacting Bose gases, *g*_0_ = 0 and we denote *g*_∞_ as *g*_max_. Equations (2) and (4) together enable quantitative predictions of macroscopic physical properties. In the following, we provide evidence for the emergence of such interaction-induced particle-hole symmetry breaking and non-mutual FES at and near a quantum critical point.

### FES in one dimension

The 1D Bose gas with delta-function interaction is an integrable model with great relevance in both theoretical and experimental contexts (see reviews [35,36]). Such gases are described by the Hamiltonian [15,37]

(5) \begin{equation*} \mathcal {H} = \sum ^N_{i=1}(-\nabla ^2_i - \mu ) + c \sum _{i \ne j} \delta (\mathbf {r}_i - \mathbf {r}_j), \end{equation*}

where *c* is the repulsive elastic interaction strength and *N* the particle number. In its dilution limit, the discrete 1D Bose-Hubbard model used in QMC simulations corresponds to Equation (5) with *c* = *U*/(2*t*^1/2^) (see Section 9 within the online supplementary material), where *U* and *t* are the onsite interaction and tunneling parameters, respectively. We exactly solve such 1D gases at the vacuum-to-TLL transition (μ_c_ = 0) [27] based on the thermodynamic Bethe ansatz (TBA) equation [37,38],

(6) \begin{eqnarray*} \varepsilon (k) &=& k^2 - \mu - \frac{T}{2\pi }\int a(k-q)\\ &&\times \,\ln(1+e^{-{\varepsilon (q)}/{T}}) \mathrm{d}q, \end{eqnarray*}

where *a*(*x*) = 2*c*/(*c*^2^ + *x*^2^), and the pressure is given by *p*(μ, *T*) = (*T*/2π)∫ln (1 + *e*^−ϵ(*k*)/*T*^)d*k*. For convenience, we present thermodynamic observables and parameters in dimensionless forms (see Section 1 within the online supplementary material). We compute the critical entropy per particle *S*_c_/*N* ≡ (*S*/*N*)(μ = μ_c_), scaled critical density $\tilde{n}_{\mathrm{c,1D}} = n_{\mathrm{c}}/T^{1/2}$ and scaled critical pressure $\tilde{p}_{\mathrm{c,1D}} = p_{\mathrm{c}}/T^{3/2}$ by numerically solving Equation (6).

**Figure 3.**
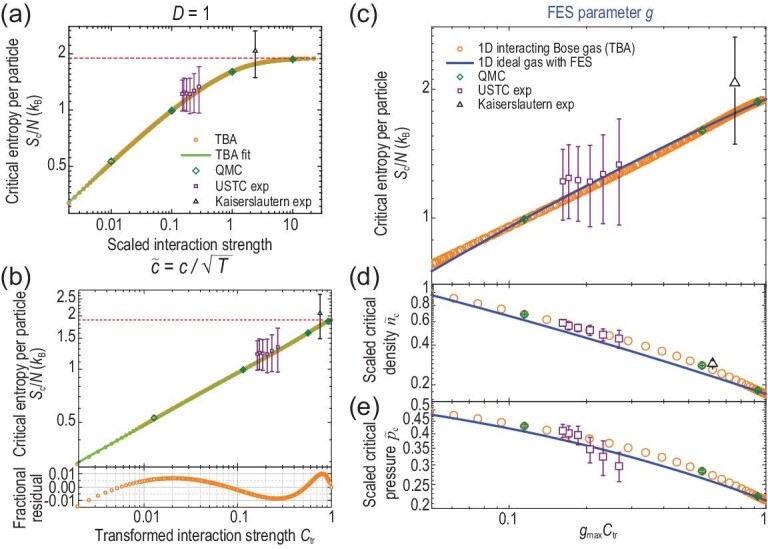
Evidence for interaction-induced FES in 1D Bose gases at a quantum critical point. (a) Critical entropy per particle *S*_c_/*N* as a function of $\tilde{c}$. Exact solutions (circles) agree excellently with QMC computations (diamonds) and agree with experiments (squares and triangles, from [27,29]). (b) Power-law scaling of *S*_c_/*N* with respect to $\mathcal {C}_{\mathrm{tr}}$. Dotted line denotes the fermionization limit *A*_∞, 1D_. (c)–(e) Under Equation (8) with *g*_max, 1D_ = 1, thermodynamic observables of interacting gases agree well with those of non-interacting quasi-particles that obey non-mutual FES: (c) *S*_c_/*N*; (d) scaled critical density $\tilde{n}_{\mathrm{c}}$; (e) scaled critical pressure $\tilde{p}_{\mathrm{c}}$. Error bars represent 1σ statistical uncertainties.

The *S*_c_/*N* increases with a scaled interaction strength $\tilde{c} = c/\sqrt{T}$ (Fig. 3(a)). It reaches *A*_∞, 1D_ ≈ 1.89738 at $\tilde{c} \rightarrow \infty$ (see Section 1 within the online supplementary material), exactly matching the *S*_c_/*N* of non-interacting fermions [37] (*g*_max, 1D_ = 1), as predicted and observed for Tonks-Girardeau gases [15,39–42]. These solutions agree with data extracted from experiments performed by the Kaiserslautern group [29] and the USTC group [27], and agree with our 1D QMC simulations (see Sections 10 and 11 within the online supplementary material).

We now verify the particle-hole symmetry breaking equation (Equation (2)) and the interaction-FES correspondence hypothesis (Equation (4)) based on *ab initio* computations (see Section 5 within the online supplementary material). We compute $\rho (\mathbf {k})$ and $\rho _{\mathrm{h}}(\mathbf {k})$ and define a $\mathbf {k}$-dependent FES parameter $g_{{\mathrm{PHSB},\mathbf {k}}}$ based on $\rho _{\mathrm{h}}(\mathbf {k})+ g_{{\mathrm{PHSB},\mathbf {k}}} \rho (\mathbf {k}) = {1}/{(2\pi )}$. We observe that $g_{{\mathrm{PHSB}, \mathbf {k}}}$ is almost homogeneous within a finite range of $|\mathbf {k}| < O(\sqrt{T})$ (see Section 5 within the online supplementary material), corresponding to low-energy elementary excitations that obey simple particle-hole symmetry breaking (Equation (2)) depicted by a non-mutual FES distribution (Equation (3)). Equation (3) associated with $g_{ {\mathrm{PHSB},\mathbf {k}=0}}$ captures the essential behaviors of $\rho (\mathbf {k})$ and $\rho _{\mathrm{h}}(\mathbf {k})$ (see Section 5 within the online supplementary material). Furthermore, the Hilbert space dimensionality ratio $R_{ \mathcal {H}\mathrm{Dim}}$ shows a proportionality relation to the interaction strength $\tilde{c}$ over a large range (Fig. 2), with a fitted coefficient $\tilde{c}_{ {1,\mathrm{PHSB}}} = 0.77(3)$. Thus, Equation (4) provides a powerful approximation that depicts the particle-hole symmetry breaking for low-energy excitations in interacting gases. We note that, based on Equation (5), particle-hole symmetry breaking can in general be depicted by $\mathbf {k}$-dependent mutual FES [14]; such mutual FES reduces to non-mutual FES under strong coupling [33], which we here derive to be for finite temperatures (see Section 5 within the online supplementary material)

(7) \begin{equation*} g^{\mathrm{strong. cpl.}}_{{\mathrm{PHSB},\mathbf {k}=0}} \approx 1-\frac{2n}{c}\bigg (1-\frac{2n}{c}\bigg )(1+e^{{\varepsilon (k=0)}/{T}}). \end{equation*}

As shown in Fig. 2, Equation (4) not only agrees with this strong-coupling analytical form, but also depicts well the numerically computed $g_{{\mathrm{PHSB},\mathbf {k}=0}}$ over a significantly larger range covering strong, intermediate and weak interactions.

The particle-hole symmetry breaking (depicted by Equations (2) and (4)) dictates the distribution functions (Equation (3)) of elementary excitations and thereby determines the macroscopic properties of interacting gases. For Bose gases, Equation (4) predicts a one-to-one mapping to non-interacting quasi-particles with FES parameter

(8) \begin{equation*} g = g_{\mathrm{max}} \mathcal {C}_{\mathrm{tr}}, \end{equation*}

where $\mathcal {C}_{\mathrm{tr}}$ is a transformed interaction parameter,

(9) \begin{equation*} \mathcal {C}_{\mathrm{tr}} \equiv \frac{\tilde{c}/\tilde{c}_1}{\tilde{c}/\tilde{c}_1+1}, \end{equation*}

and *g*_max, 1D_ = 1. Based on the computed critical entropy per particle, we observe two scaling functions (Fig. 3(b) and (c)) that are similar to each other, which is characteristic of the interaction-FES correspondence (Equation (8)). For non-interacting FES quasi-particles, the *S*_c, FES_/*N* at μ_c_ = 0 exhibits a power-law scaling with respect to *g* (Fig. 3(c), blue curve): $S_{\mathrm{c,FES}}/N = A_{\infty ,\mathrm{1D}} g^{\beta _{\mathrm{FES,1D}}}$, with β_FES, 1D_ = 0.298(2) fitted for 0.05 < *g* ≤ 1. This scaling suggests, and we indeed observe, that the *S*_c_/*N* for interacting Bose gases accordingly obeys a power-law scaling with respect to $\mathcal {C}_{\mathrm{tr}}$ (Fig. 3(b)):

(10) \begin{equation*} S_{\mathrm{c}}/N = A_{\infty ,\mathrm{1D}} \mathcal {C}_{\mathrm{tr}}^{\beta _{\mathrm{1D}}}. \end{equation*}

Here β_1D_ = 0.298(1) and $\tilde{c}_{1} = 0.772(5)$ are fitted parameters. Under Equation (8), these two scaling functions match each other, and the numerical data agree within 4% (Fig. 3(c)). We note that Equation (10) agrees excellently with exact solutions within $1\%$ over $0.002 < \tilde{c} < \infty$, and the precisely determined $\tilde{c}_1$ conforms well to the $\tilde{c}_{{1,\mathrm{PHSB}}}$ determined earlier. Thus, identifying the power-law scaling for *S*_c_/*N* provides a smoking-gun signature and a precise determination of the interaction-FES correspondence.

We find agreement within $15\%$ and $8\%$ for $\tilde{n}_{\mathrm{c}}$ and $\tilde{p}_{\mathrm{c}}$, respectively (Fig. 3(d) and (e)). The overall good agreement for *S*_c_/*N*, $\tilde{n}_{\mathrm{c}}$ and $\tilde{p}_{\mathrm{c}}$ shows that macroscopic thermodynamic observables are determined by interaction-induced particle-hole symmetry breaking. Therefore, these observables can serve as a practical gauge for conveniently measuring the corresponding non-mutual FES, especially when *ab initio* computations are difficult or unavailable.

### FES in two dimensions

In higher dimensions, while the Bethe ansatz in general does not apply, particle-hole symmetry breaking remains a key characteristic [43] that governs measurable macroscopic properties of interacting gases. Here we benchmark the applicability of Equations (2) and (4) in 2D gases. Using QMC simulations [44,45], we study a 2D Bose-Hubbard lattice gas that has a vacuum-to-superfluid quantum phase transition at μ_c_ = −4*t* [28]. The Bose-Hubbard Hamiltonian is given by

(11) \begin{eqnarray*} \hat{H} = -t \sum _{\langle i,j\rangle }(\hat{b}_{i}^\dagger \hat{b}_{j}^{}\! +\hat{b}_{j}^\dagger \hat{b}_{i})\\ +\sum _{i}\bigg [\frac{U}{2}\hat{n}_i(\hat{n}_{i}-1)-\mu \hat{n}_i\bigg ], \end{eqnarray*}

where $\hat{b}_{i}^{\dagger}$ and $\hat{b}_{i}$ are the creation and annihilation operators at site *i*, $\hat{n}_{i} = \hat{b}_{i}^\dagger \hat{b}_{i}$ and 〈*i, j*〉 runs over all nearest neighboring sites. We define a scaled interaction strength $\tilde{c}_{\mathrm{2D}} = U/(2t)$ (see Section 9 within the online supplementary material) that is the lattice-gas equivalence [28,31] of the interaction parameter $\sqrt{8\pi }a/l_z$ for 2D Bose gases without lattices, where *a* is the scattering length and *l_z_* is an oscillator length [32].

To obtain physical properties that are insensitive to the lattice structure, we perform QMC simulations for each $\tilde{c}_{\mathrm{2D}}$ at a series of temperatures down to *T* = 0.1*t*. We extract scaled quantities *S*_c_/*N*, $\tilde{n}_{\mathrm{c,2D}} = n_{\mathrm{c}}/T$ and $\tilde{p}_{\mathrm{c,2D}} = p_{\mathrm{c}}/T^2$ for each *T*, and perform extrapolation towards *T* = 0 for each quantity (see Section 10 within the online supplementary material). We test this extrapolation protocol on a 1D Bose-Hubbard system (see Section 10 within the online supplementary material) and find excellent agreement with exact solutions (Fig. 3).

**Figure 4.**
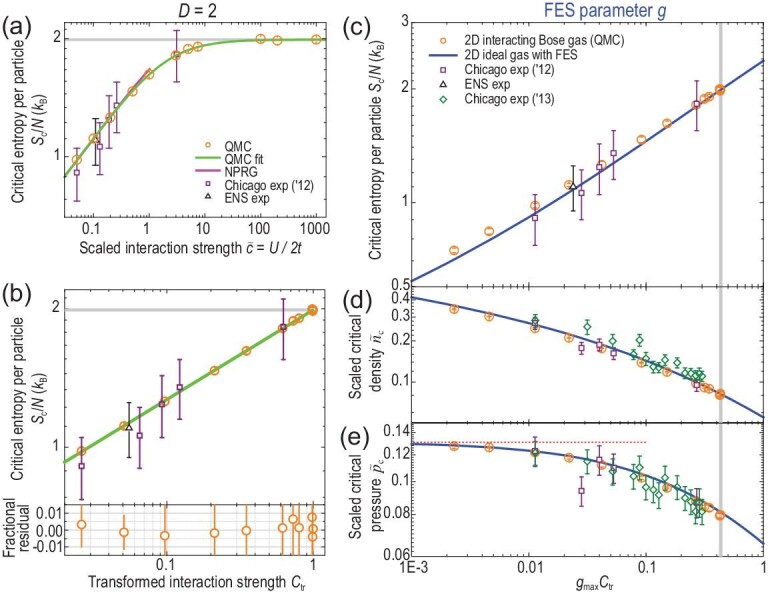
Evidence for interaction-induced FES in 2D Bose gases at a quantum critical point. (a) Critical entropy per particle *S*_c_/*N* as a function of $\tilde{c}_{\mathrm{2D}}$. QMC results (circles) agree with NPRG computations [46] and experiments [28,30]. (b) Power-law scaling of *S*_c_/*N* with respect to $\mathcal {C}_{\mathrm{tr}}$. (c)–(e) Given Equation (8) with *g*_max, 2D_ = 0.432(14), thermodynamic observables of interacting gases agree well with those of non-interacting quasi-particles that obey non-mutual FES: (c) *S*_c_/*N*; (d) scaled critical density $\tilde{n}_{\mathrm{c}}$; (e) scaled critical pressure $\tilde{p}_{\mathrm{c}}$, with the dotted line denoting the non-interacting boson limit $\tilde{p}_{\mathrm{c}0} = {\pi }/{24}$ [46]. Our results agree with existing experiments [28,30,31]. Horizontal and vertical gray bands mark *A*_∞, 2D_ and *g*_max, 2D_, respectively. Error bars represent 1σ statistical uncertainties.

In two dimensions, we identify the same interaction-FES correspondence (Equation (8)) as in one dimension. The *S*_c_/*N* increases with $\tilde{c}_{\mathrm{2D}}$ and reaches *A*_∞, 2D_ = 1.988(14) at $\tilde{c}_{\mathrm{2D}} = \infty$ (Fig. 4(a)), matching the *S*_c, FES_/*N* of non-interacting FES quasi-particles with *g*_max, 2D_ = 0.432(14). Our QMC data agree well with a non-perturbative renormalization group (NPRG) computation [46], and with experiments by the Chicago [28] and ENS [30] groups. Based on Equation (9) and $\tilde{c}_{1,\mathrm{2D}} = 1.9(3)$, *S*_c_/*N* shows an excellent power-law scaling with respect to $\mathcal {C}_{\mathrm{tr}} ={(\tilde{c}_{\mathrm{2D}}/\tilde{c}_{ {1,\mathrm{2D}}})} / {(\tilde{c}_{ \mathrm{2D}}/\tilde{c}_{ {1,\mathrm{2D}}} + 1)}$ (Fig. 4(b)):

(12) \begin{equation*} S_{\mathrm{c}}/N = A_{\infty ,\mathrm{2D}} \mathcal {C}_{\mathrm{tr}}^{\beta _{\mathrm{2D}}} \end{equation*}

with *A*_∞, 2D_, $\tilde{c}_{1,\mathrm{2D}}$, and β_2D_ = 0.20(1) fitted for $0.05 \le \tilde{c}_{\mathrm{2D}}<\infty$ ($0.026 \le \mathcal {C}_{\mathrm{tr}} \le 1$). We then test the predictions of Equations (2) and (4) (with *g*_max, 2D_ and $\tilde{c}_{1,\mathrm{2D}}$ determined above) using the QMC data.

We find evidence for emergent non-mutual FES based on good agreement for *S*_c_/*N*, $\tilde{n}_{\mathrm{c}}$ and $\tilde{p}_{\mathrm{c}}$. The FES quasi-particles also exhibit a power-law scaling, $S_{\mathrm{c,FES}}/N = A_{\mathrm{FES, 2D}} g^{\beta _{\mathrm{FES,2D}}}$, with *A*_FES, 2D_ ≡ (*S*_c, FES_/*N*)(*g* = 1) ≈ 2.373. The exponent β_FES, 2D_ = 0.2122(1) is fitted for 0.02 ≤ *g* ≤ 1 and agrees with β_2D_. Given Equation (8), the two power-law scaling functions for *S*_c_/*N* versus $g_{\mathrm{max, 2D}} \mathcal {C}_{\mathrm{tr}}$ and for *S*_c, FES_/*N* versus *g* agree well within $5\%$ (Fig. 4(c)). Accordingly, $\tilde{n}_{\mathrm{c,2D}}$ and $\tilde{p}_{\mathrm{c,2D}}$ show agreement within $5\%$ and $3\%$, respectively (Fig. 4(d) and (e)). Our simulations agree with existing experiments [28,30,31] at and near the quantum critical point (see also Fig. S5 within the online supplementary material). These numerical and experimental data together are fully consistent with, and thereby strongly support, the emergence of interaction-induced particle-hole symmetry breaking and non-mutual FES in two dimensions (Equations (2) and (8)). The less-than-unity *g*_max, 2D_ = 0.432(14) shows incomplete fermionization of strongly interacting 2D Bose gases.

In summary, we established a generic connection between particle-hole symmetry breaking and FES and then found strong evidence for interaction-induced non-mutual FES at and near a quantum critical point. Our non-perturbative approach holds promise for studying the dynamical evolutions [24], transport properties [25] and hydrodynamics [47] of quantum systems in arbitrary dimensions, and for studying other integrable models such as multi-component systems. In particular, such simple non-mutual FES is expected to emerge in the charge degree of freedom at the quantum critical region of the multi-component ultracold atoms in one and higher dimensions, whereas the spin degrees of freedom are frozen out at quantum criticality. Moreover, our approach provides a route to understanding strongly interacting quantum materials where experiments can be both enriched and complicated by inelastic collisional losses and finite temperature effects [31,48,49].

## METHODS

### Quantum Monte Carlo simulations

In our work, we apply the worm algorithm in the path-integral representation to simulate the Bose-Hubbard model in both one and two dimensions using the QMC method. Here, we mainly focus on three observables: particle density *n*, pressure *p* and entropy per particle *S*/*N*. Our QMC data are obtained by spending about 2 × 10^5^ CPU hours. Further details of the QMC simulation can be found in Sections 8 to 11 within the online supplementary material.

### Statistical methods for data analysis

The error bars in figures and texts represent 1σ statistical uncertainties of the QMC simulations or experimental measurements. Based on a set of numerical or experimental data and a specific model, a least-square fit can be performed to determine the best fitting parameters as well as the standard errors of these parameters.

### DATA AVAILABILITY

The data supporting the findings of this study are available within the paper and accompanying online supplementary material.

## Supplementary Material

nwac027_Supplemental_File

## References

[1] Khare A . Fractional Statistics and Quantum Theory. Singapore: World Scientific Publishing, 2005. 10.1142/5752

[2] Leinaas JM , Myrheim J. On the theory of identical particles. Nuovo Cim B 1977; 37: 1–23. 10.1007/BF02727953

[3] Wilczek F . Magnetic flux, angular momentum, and statistics. Phys Rev Lett 1982; 48: 1144–6. 10.1103/PhysRevLett.48.1144

[4] Wilczek F . Quantum mechanics of fractional-spin particles. Phys Rev Lett 1982; 49: 957–9. 10.1103/PhysRevLett.49.957

[5] Arovas D , Schrieffer JR, Wilczek F. Fractional statistics and the quantum Hall effect. Phys Rev Lett 1984; 53: 722–3. 10.1103/PhysRevLett.53.722

[6] Laughlin RB . Superconducting ground state of noninteracting particles obeying fractional statistics. Phys Rev Lett 1988; 60: 2677–80. 10.1103/PhysRevLett.60.2677 10038420

[7] Laughlin RB . The relationship between high-temperature superconductivity and the fractional quantum Hall effect. Science 1988; 242: 525–33. 10.1126/science.242.4878.525 17815892

[8] Haldane FDM . Fractional statistics in arbitrary dimensions: a generalization of the Pauli principle. Phys Rev Lett 1991; 67: 937–40. 10.1103/PhysRevLett.67.937 10045028

[9] Wu YS . Statistical distribution for generalized ideal gas of fractional-statistics particles. Phys Rev Lett 1994; 73: 922–5. 10.1103/PhysRevLett.73.922 10057575

[10] Isakov SB . Statistical mechanics for a class of quantum statistics. Phys Rev Lett 1994; 73: 2150–3. 10.1103/PhysRevLett.73.2150 10056985

[11] Calogero F . Solution of a three-body problem in one dimension. J Math Phys 1969; 10: 2191–6. 10.1063/1.1664820

[12] Calogero F . Ground state of a one-dimensional *n*-body system. J Math Phys 1969; 10: 2197–200. 10.1063/1.1664821

[13] Sutherland B . Quantum many-body problem in one dimension: thermodynamics. J Math Phys 1969; 12: 251–6. 10.1063/1.1665585

[14] Bernard D , Wu YS. A note on statistical interactions and the thermodynamic Bethe ansatz. In: Ge ML and Wu YS (eds.). *Proceedings of the 6th Nankai Workshop on “New Developments of Integrable Systems and Long-Range Interaction Models*”. Singapore: World Scientific Publishing, 1995.

[15] Lieb EH , Liniger W. Exact analysis of an interacting Bose gas. I. The general solution and the ground state. Phys Rev 1963; 130: 1605–16. 10.1103/PhysRev.130.1605

[16] Kundu A . Exact solution of double delta function bose gas through an interacting anyon gas. Phys Rev Lett 1999; 83: 1275–8. 10.1103/PhysRevLett.83.1275

[17] Batchelor MT , Guan XW, Oelkers N. One-dimensional interacting anyon gas: low-energy properties and Haldane exclusion statistics. Phys Rev Lett 2006; 96: 210402. 10.1103/PhysRevLett.96.210402 16803221

[18] Ha ZNC . Exact dynamical correlation functions of Calogero-Sutherland model and one-dimensional fractional statistics. Phys Rev Lett 1994; 73: 1574–7. 10.1103/PhysRevLett.73.1574 10056829

[19] Hashimoto M , He RH, Tanaka K et al. Particle-hole symmetry breaking in the pseudogap state of Bi2201. Nat Phys 2010; 6: 414–8. 10.1038/nphys1632

[20] Miller TL , Zhang W, Eisaki H et al. Particle-hole asymmetry in the cuprate pseudogap measured with time-resolved spectroscopy. Phys Rev Lett 2017; 118: 097001. 10.1103/PhysRevLett.118.097001 28306293

[21] Zhang Y , Wojs A, Jain JK. Landau-level mixing and particle-hole symmetry breaking for spin transitions in the fractional quantum Hall effect. Phys Rev Lett 2016; 117: 116803. 10.1103/PhysRevLett.117.116803 27661711

[22] Bhaduri RK , Murthy MVN, Srivastava MK. Fermions at unitarity and Haldane exclusion statistics. J Phys B 2007; 40: 1775–80. 10.1088/0953-4075/40/10/012

[23] Tabert CJ , Carbotte JP. Particle-hole asymmetry in gapped topological insulator surface states. Phys Rev B 2015; 91: 235405. 10.1103/PhysRevB.91.235405

[24] Balakrishnan R , Satija II, Clark CW. Particle-hole asymmetry and brightening of solitons in a strongly repulsive Bose-Einstein condensate. Phys Rev Lett 2009; 103: 230403. 10.1103/PhysRevLett.103.230403 20366132

[25] Demchenko DO , Joura AV, Freericks JK. Effect of particle-hole asymmetry on the Mott-Hubbard metal-insulator transition. Phys Rev Lett 2004; 92: 216401. 10.1103/PhysRevLett.92.216401 15245299

[26] Kusunose H , Miyake K, Shimizu Y et al. Numerical renormalization-group study of particle-hole symmetry breaking in two-channel Kondo problem: effect of repulsion among conduction electrons and potential scattering. Phys Rev Lett 1996; 76: 271–4. 10.1103/PhysRevLett.76.271 10061059

[27] Yang B , Chen YY, Zheng YG et al. Quantum criticality and the Tomonaga-Luttinger liquid in one-dimensional Bose gases. Phys Rev Lett 2017; 119: 165701. 10.1103/PhysRevLett.119.165701 29099230

[28] Zhang X , Hung CL, Tung SK et al. Observation of quantum criticality with ultracold atoms in optical lattices. Science 2012; 335: 1070–2. 10.1126/science.1217990 22345397

[29] Vogler A , Labouvie R, Stubenrauch F et al. Thermodynamics of strongly correlated one-dimensional Bose gases. Phys Rev A 2013; 88: 031603(R). 10.1103/PhysRevA.88.031603

[30] Yefsah T , Desbuquois R, Chomaz L et al. Exploring the thermodynamics of a two-dimensional Bose gas. Phys Rev Lett 2011; 107: 130401. 10.1103/PhysRevLett.107.130401 22026829

[31] Ha LC , Hung CL, Zhang X et al. Strongly interacting two-dimensional Bose gases. Phys Rev Lett 2013; 110: 145302. 10.1103/PhysRevLett.110.145302 25167003

[32] Hung CL , Zhang X, Gemelke N et al. Observation of scale invariance and universality in two-dimensional Bose gases. Nature 2011; 470: 236–40. 10.1038/nature09722 21270797

[33] Batchelor MT , Guan XW. Fermionization and fractional statistics in the strongly interacting one-dimensional Bose gas. Laser Phys Lett 2007; 4: 77–83. 10.1002/lapl.2006100681

[34] Sachdev S , Demler E. Competing orders in thermally fluctuating superconductors in two dimensions. Phys Rev B 2004; 69: 144504. 10.1103/PhysRevB.69.144504

[35] Cazalilla MA , Citro R, Giamarchi T et al. One dimensional bosons: from condensed matter systems to ultracold gases. Rev Mod Phys 2011; 83: 1405–66. 10.1103/RevModPhys.83.1405

[36] Batchelor MT , Foerster A. Yang-Baxter integrable models in experiments: from condensed matter to ultracold atoms. J Phys A: Math Theor 2016; 49: 173001. 10.1088/1751-8113/49/17/173001

[37] Yang CN , Yang CP. Thermodynamics of a one-dimensional system of bosons with repulsive delta-function interaction. J Math Phys 1969; 10: 1115–22. 10.1063/1.1664947

[38] Takahashi M . Thermodynamics of One-Dimensional Solvable Models. Cambridge: Cambridge University Press, 1999.

[39] Tonks L . The complete equation of state of one, two and three-dimensional gases of hard elastic spheres. Phys Rev 1936; 50: 955–63. 10.1103/PhysRev.50.955

[40] Girardeau M . Relationship between systems of impenetrable bosons and fermions in one dimension. J Math Phys 1960; 1: 516–23. 10.1063/1.1703687

[41] Paredes B , Widera A, Murg V et al. Tonks-Girardeau gas of ultracold atoms in an optical lattice. Nature 2004; 429: 277–81. 10.1038/nature02530 15152247

[42] Kinoshita T , Wenger T, Weiss DS. Observation of a one-dimensional Tonks-Girardeau gas. Science 2004; 305: 1125–8. 10.1126/science.1100700 15284454

[43] Bhaduri RK , Murthy MVN, Srivastava MK. Fractional exclusion statistics and two dimensional electron systems. Phys Rev Lett 1996; 76: 165–8. 10.1103/PhysRevLett.76.165 10061032

[44] Prokof’ev N , Svistunov B, Tupitsyn I. Worm algorithm in quantum Monte Carlo simulations. Phys Lett A 1998; 238: 253–7. 10.1103/PhysRevE.74.036701

[45] Prokof’ev N , Svistunov B, Tupitsyn I. Exact, complete, and universal continuous-time worldline Monte Carlo approach to the statistics of discrete quantum systems. J Exp Theor Phys 1998; 87: 310–21. 10.1134/1.558661

[46] Rancon A , Dupuis N. Universal thermodynamics of a two-dimensional Bose gas. Phys Rev A 2012; 85: 063607. 10.1103/PhysRevA.85.063607

[47] Nardis JD , Bernard D, Doyon B. Hydrodynamic diffusion in integrable systems. Phys Rev Lett 2018; 121: 160603. 10.1103/PhysRevLett.121.160603 30387673

[48] Fletcher RJ , Gaunt AL, Navon N et al. Stability of a unitary Bose gas. Phys Rev Lett 2013; 111: 125303. 10.1103/PhysRevLett.111.125303 24093273

[49] Eismann U , Khaykovich L, Laurent S et al. Universal loss dynamics in a unitary Bose gas. Phys Rev X 2016; 6: 021025. 10.1103/PhysRevX.6.021025

