# Supplementary material for: Interaction-induced particle-hole symmetry breaking and fractional exclusion statistics
Source: Natl Sci Rev. 2022 Feb 24;9(12):nwac027. doi: 10.1093/nsr/nwac027 (PMC9885437; doi:10.1093/nsr/nwac027)
Supplement: nwac027_Supplemental_File [file nwac027_supplemental_file.pdf]

Supplementary Data for

Interaction-induced particle-hole symmetry breaking and fractional exclusion statistics

Xibo Zhang*, Yang-Yang Chen, Longxiang Liu, Youjin Deng, and Xiwen Guan

*Corresponding author. xibo@pku.edu.cn (X.Z.)

This PDF file includes:

Supplemental Text

Figs. S1 to S12

References 50-56, following References 1–49 in the main text

Contents

1 Yang-Yang thermodynamic Bethe ansatz equation 3

2 Fractional Exclusion Statistics 7

3 From correlated to nearly independent quasi-momentum cells: the essence of FES 9

4 Particle-hole symmetry breaking and the FES distribution 15

5 An ab initio computation: the emergence of simple, non-mutual FES for the low-energy excitations in 1D interacting gases 18

6 Emergence of interaction-induced non-mutual FES in the strongly correlated regime at and near the quantum critical point 23

7 Comparison between Yang-Yang equation and FES near the quantum critical point 26

8 Dimension analysis and dimensionless quantities 30

9 Mapping between Bose gases and the discrete Bose-Hubbard model 33

10 Extrapolation towards zero temperature 36

11 Measuring of the observables in Bose-Hubbard model 43

12 Comparing simulations with existing experimental measurements 51

1 Yang-Yang thermodynamic Bethe ansatz equation

The thermodynamic properties of δ-function interacting Bose gases in one dimension (1D) can be obtained by solving Yang-Yang thermodynamic Bethe ansatz equation (TBAE)[37]

$$\varepsilon(k) = \frac{\hbar^2 k^2}{2m} - \mu - \frac{k_B T}{2\pi} \int_{-\infty}^{\infty} \frac{2c}{c^2 + (k-q)^2} \ln\left(1 + e^{-\frac{\varepsilon(q)}{k_B T}}\right) dq, \tag{S1}$$

where $\varepsilon(k)$ is called "dressed energy", k is quasi-momentum, μ is chemical potential, T is temperature and $c = -2/a_{\rm 1D}$ with $a_{\rm 1D}$ being 1D scattering length. Thus the grand thermodynamic potential of unit length, namely, pressure p can be obtained by

$$p = \frac{k_B T}{2\pi} \int_{-\infty}^{\infty} \ln\left(1 + e^{-\frac{\varepsilon(k)}{k_B T}}\right) dk. \tag{S2}$$

Other thermodynamic properties, such as particle density n, entropy density s are given by the derivatives of the pressure, namely,

$$n = \frac{\partial p}{\partial \mu}|_{c,T}, \quad s = \frac{\partial p}{\partial T}|_{c,\mu} \tag{S3}$$

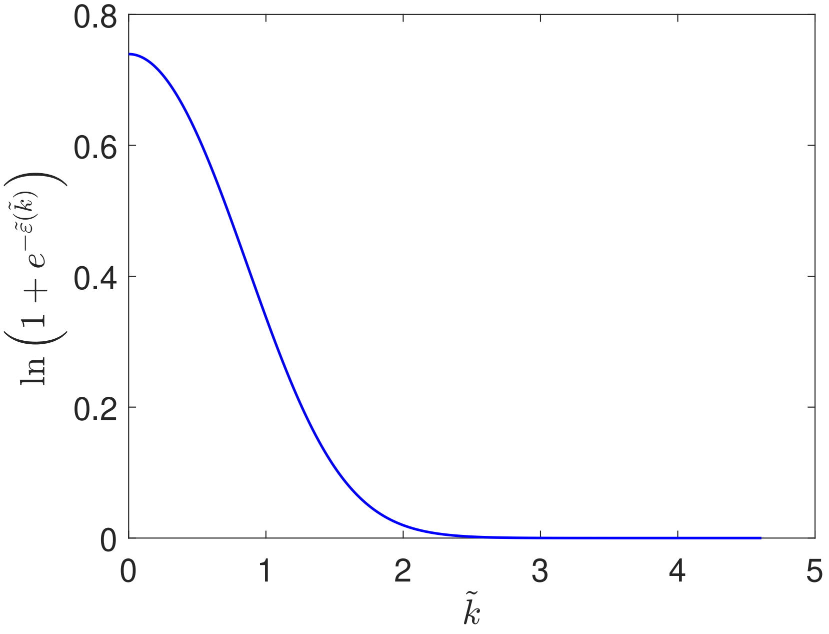

Figure S1: $\ln\left(1+e^{-\tilde{\varepsilon}(\tilde{k})}\right)$ vs $\tilde{k}$. Here we take $\tilde{\mu}=0, \tilde{c}=5$ and the cutoff $\tilde{k}_c \approx 4.6$.

For our convenience in analysis of critical phenomenon, we define the following dimensionless parameters

$$\tilde{k} \equiv \frac{\hbar k}{\sqrt{2mk_BT}}, \quad \tilde{c} \equiv \frac{\hbar c}{\sqrt{2mk_BT}}, \quad \tilde{\mu} = \frac{\mu}{k_BT}, \quad \tilde{\varepsilon} \equiv \frac{\varepsilon}{k_BT} \tag{S4}$$

and the dimensionless thermodynamic properties

$$\tilde{p} \equiv \frac{\hbar p}{k_BT\sqrt{2mk_BT}}, \quad \tilde{n} = \frac{\hbar n}{\sqrt{2mk_BT}}, \quad \tilde{s} \equiv \frac{\hbar s}{k_B\sqrt{2mk_BT}}. \tag{S5}$$

Consequently, the dimensionless Yang-Yang TBAE is given by

$$\tilde{\varepsilon}(\tilde{k}) = \tilde{k}^2 - \tilde{\mu} - \frac{1}{2\pi}\int_{-\infty}^{\infty} \frac{2\tilde{c}}{\tilde{c}^2 + (\tilde{k}-\tilde{q})^2} \ln\left(1+e^{-\tilde{\varepsilon}(\tilde{q})}\right) d\tilde{q}. \tag{S6}$$

This serves as an equation of states for a whole temperature regime. This integral equation can be numerically solved by iteration method. In order to make a discretization in the variable space $\tilde{k}$, we need to find a proper cutoff $\tilde{k}_c$. The cutoff $\tilde{k}_c$ is determined by choosing $\ln(1 + e^{-\tilde{\varepsilon}(\tilde{k}_c)}) < 10^{-9}$ because this term decreases quickly with increasing $\tilde{k}$, as it is shown in the Fig. S1. The number of discretization from $\tilde{k} = 0$ to $\tilde{k} = \tilde{k}_c$ is $N_k = 1000$. Once we get $\tilde{\varepsilon}(\tilde{k})$ the dimensionless pressure $\tilde{p}$ can be obtained by

$$\tilde{p} \approx \frac{1}{2\pi}\int_{-\tilde{k}_c}^{\tilde{k}_c} \ln\left(1+e^{-\tilde{\varepsilon}(\tilde{k})}\right) d\tilde{k}. \tag{S7}$$

Furthermore, we compare the pressure by taking different N_k and find the difference can be negligible, as shown in the Fig.S2.

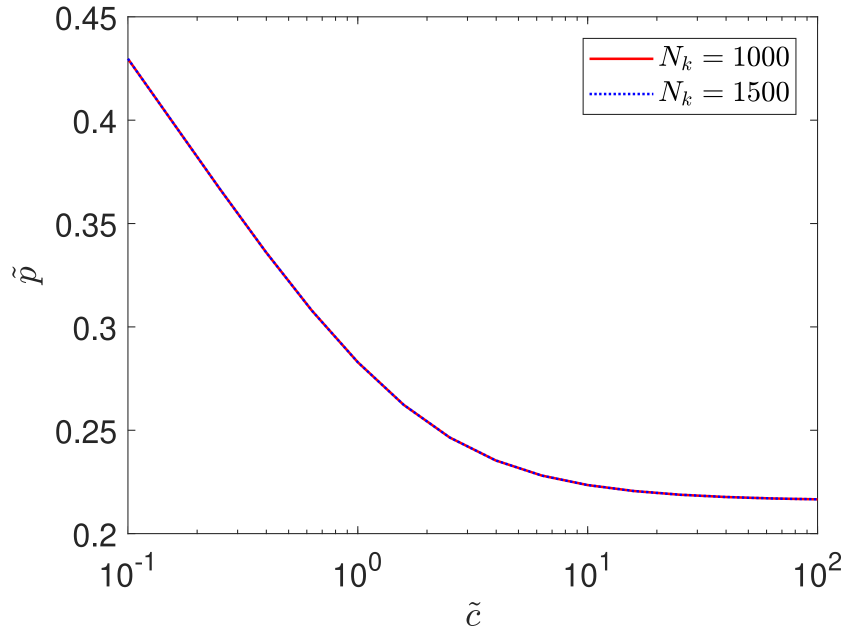

Figure S2: The dimensionless pressure $\tilde{p}$ vs $\tilde{c}$. The red solid line corresponds to the number of discretization $N_k = 1000$ and the blue dot line corresponds to $N_k = 1500$.

Based on the above numerical method, the density $\tilde{n}$ and entropy $\tilde{s}$ are obtained by the derivatives of pressure [50]

$$\begin{aligned}
\tilde{n} &\approx - \frac{1}{2\pi}\int_{-\tilde{k}_c}^{\tilde{k}_c} \frac{1}{1+e^{\tilde{\varepsilon}(\tilde{k})}}\varepsilon_\mu d\tilde{k}, && \text{(S8)}\\
\tilde{s} &\approx \tilde{p} - \frac{1}{2\pi}\int_{-\tilde{k}_c}^{\tilde{k}_c} \frac{1}{1+e^{\tilde{\varepsilon}(\tilde{k})}}\left(\varepsilon_T - \tilde{\varepsilon}(\tilde{q})\right) dk, && \text{(S9)}
\end{aligned}$$

where the derivatives of $\varepsilon_\mu \equiv \frac{\partial\varepsilon(k)}{\partial\mu}, \varepsilon_T \equiv \frac{\partial\varepsilon(k)}{\partial T}$ are given by

$$\begin{aligned}
\varepsilon_\mu &\approx -1 + \frac{1}{2\pi}\int_{-\tilde{k}_c}^{\tilde{k}_c} \frac{2\tilde{c}}{\tilde{c}^2+(\tilde{k}-\tilde{q})^2}\frac{1}{e^{\tilde{\varepsilon}(\tilde{q})}+1}\varepsilon_\mu d\tilde{q}, && \text{(S10)}\\
\varepsilon_T &\approx \tilde{\varepsilon}(\tilde{k}) - \tilde{k}^2 + \tilde{\mu} + \frac{1}{2\pi}\int_{-\tilde{k}_c}^{\tilde{k}_c} \frac{2\tilde{c}}{\tilde{c}^2+(\tilde{k}-\tilde{q})^2}\frac{1}{e^{\tilde{\varepsilon}(\tilde{q})}+1}\left(\varepsilon_T - \tilde{\varepsilon}(\tilde{q})\right) d\tilde{q}. && \text{(S11)}
\end{aligned}$$

These two equations may be numerically solved by iteration.

As an example, when $\tilde{c}$ approaches $+\infty$, the last integral term in the dimensionless TBA, Eq. S6, can be ignored and the gas behaves like free fermions [37]. In particular, at the critical point $\tilde{\mu} = 0$, the entropy per particle can be computed analytically:

$$\frac{S_{\mathrm{c}}}{N} = \frac{3}{2}\frac{\mathrm{Li}_{3/2}(-1)}{\mathrm{Li}_{1/2}(-1)} \approx 1.89738, \qquad \text{(S12)}$$

where Li_s is a polylogarithmic function of order s.

2 Fractional Exclusion Statistics

In 1991, Haldane [8] formulated a description of the fractional exclusion statistics (FES) based on a generalized Pauli exclusion principle. This FES was further formulated by Wu [9,14] and

others [10,18]. It has been proved that the 1D δ-function interacting Bose gas can be mapped onto ideal particles with FES, see [9,14,33]. In this sense, the dynamical and statistical interactions are transmutable. This allows one to deal with the thermodynamic properties through Haldane's FES. In general, the relation between the interaction strength and FES parameter is very complicated. However, under a strong interaction strength, the system may be equivalent to an ideal gas with a non-mutual FES, i.e. the FES parameter does not depends on the momenta of particles. For such a non-mutual FES with a parameter g, the occupation number f in a state with energy $\epsilon = \frac{\hbar^2 \boldsymbol{k}^2}{2m}$ is given by

$$f(\epsilon) = \frac{1}{w+g}, \tag{S13}$$

where w obeys

$$w^g (1+w)^{1-g} = e^{\frac{\epsilon-\mu}{k_B T}} = e^{\tilde{\boldsymbol{k}}^2 - \tilde{\mu}}. \tag{S14}$$

The thermodynamic properties in D dimension, such as pressure p, energy density E, and particle density n are given by

$$p = \frac{k_B T}{(2\pi)^D} \int_{-\infty}^{\infty} \ln \frac{1+w}{w} d^D \boldsymbol{k}, \tag{S15}$$

$$E = \frac{1}{(2\pi)^D} \int_{-\infty}^{\infty} \frac{\epsilon}{w+g} d^D \boldsymbol{k}, \tag{S16}$$

$$n = \frac{1}{(2\pi)^D} \int_{-\infty}^{\infty} \frac{1}{w+g} d^D \boldsymbol{k}, \tag{S17}$$

and the entropy density s can be obtained from thermodynamic relation $E = -p + \mu n + sT$.

The left hand side of Eq. S14 is monotonically increasing with w. For a given g, $\tilde{\mu}$ and $\tilde{\boldsymbol{k}}$, Eq.(S14) can be numerically solved by bisection method. The relative error of w in our

numerical calculation is less than 10^{-6}. The cutoff $\tilde{k}_c$ and discrete number N_k here are the same with the one for solving Yang-Yang equation.

3 From correlated to nearly independent quasi-momentum cells: the essence of FES

As described in the main text, in a quantum many-body system, interaction dresses the constituent particles to form quasi-particles that are statistically distributed over the quasi-momentum space. In each quasi-momentum cell that defines the species in Haldane's FES definition (Eq. 1 in the main text), the number of unoccupied states depends on the numbers of occupied states in the same cell and in other cells, forming a net of correlated cells in general. Such correlated cells can be depicted by quasi-momentum-dependent particle-hole symmetry breaking; see Eq. S43. In this section, we demonstrate a duality relation between the equal-time two-point correlation function in the real space ($\mathbf{r}$ space) and a corresponding two-point correlator in the quasi-momentum space ($\mathbf{k}$ space). Such $\mathbf{r}-\mathbf{k}$ duality relation suggests that under certain conditions when the system is strongly correlated with large characteristic lengths in the real space, the quasi-momentum cells become nearly independent in the quasi-momentum space.

We discuss the equal-time two-point correlation function for a physical quantity f in a homogeneous D-dimensional sample. The fluctuation of f at position r is given by

$$\delta f(\mathrm{r}) \equiv f(\mathrm{r}) - \langle f(\mathrm{r})\rangle \equiv f(\mathrm{r}) - \bar{f}, \tag{S18}$$

where <> denotes the ensemble averaging. In addition, we assume the system satisfies a certain

form of translational invariance such that the two-point correlation function is a function of the position difference only:

$$\langle \delta f(\mathrm{r}_1)\delta f(\mathrm{r}_2)\rangle \;=\; \bar{f}G_2(\mathrm{r}_2-\mathrm{r}_1). \tag{S19}$$

For convenience of derivations, we define the position on discrete lattice sites in a finite and large D-dimensional cubic region with periodic boundary conditions for the system. Along each dimension, the minimum spacing d_r and the maximum length $L_r = 2Md_r$ corresponds to a maximum quasi-momentum value $L_k = \frac{2\pi}{d_r}$ and a minimum spacing $d_k = \frac{2\pi}{L_r} = \frac{L_k}{2M}$ in the quasi-momentum space, where the ranges of real-space coordinates and quasi-momentum coordinates are $[-L_r/2, L_r/2]$ and $[-L_k/2, L_k/2]$, respectively. Here, $2M+1$ and $(2M+1)^D$ is the number of lattice sites along one dimension and the total number of lattice sites in the whole region, respectively. We denote $V = L_r^D$ to be the volume of the D-dimensional region in the real space.

We define the Fourier transform of the r-space fluctuation ($\delta f(\mathrm{r})$) to be

$$\delta\tilde{f}(\mathrm{k}) \;=\; \sum_{\mathrm{r}} d_r^D e^{-\mathbb{I}\mathrm{k}\cdot\mathrm{r}}\delta f(\mathrm{r}), \tag{S20}$$

where $\mathbb{I}$ is the unit of imaginary number. We now compute the equal-time two-point correlation function *for the above-defined Fourier components* in the quasi-momentum space:

$$\begin{aligned}\left\langle \delta\tilde{f}(\mathrm{k}_1)\delta\tilde{f}(\mathrm{k}_2)\right\rangle &= \sum_{\mathrm{r}_1,\mathrm{r}_2} d_r^{2D} e^{-\mathbb{I}(\mathrm{k}_1\cdot\mathrm{r}_1+\mathrm{k}_2\cdot\mathrm{r}_2)}\left\langle \delta f(\mathrm{r}_1)\delta f(\mathrm{r}_2)\right\rangle \\ &= \sum_{\mathrm{r}_1,\mathrm{r}_2} d_r^{2D} e^{-\mathbb{I}(\mathrm{k}_1\cdot\mathrm{r}_1+\mathrm{k}_2\cdot\mathrm{r}_2)}\bar{f}G_2(\mathrm{r}_2-\mathrm{r}_1)\end{aligned} \tag{S21}$$

Let $\bar{\mathrm{r}} \equiv (\mathrm{r}_1 + \mathrm{r}_2)/2$, $\Delta\mathrm{r} \equiv \mathrm{r}_2 - \mathrm{r}_1$, $\bar{\mathrm{k}} \equiv (\mathrm{k}_1 + \mathrm{k}_2)/2$, and $\Delta\mathrm{k} \equiv \mathrm{k}_2 - \mathrm{k}_1$, we notice that

$$\mathrm{k}_1 \cdot \mathrm{r}_1 + \mathrm{k}_2 \cdot \mathrm{r}_2 \;=\; 2\bar{\mathrm{k}} \cdot \bar{\mathrm{r}} + \frac{1}{2}\Delta\mathrm{k} \cdot \Delta\mathrm{r} \tag{S22}$$

Thus

$$\begin{aligned}
\left\langle \delta\tilde{f}(\mathrm{k}_1)\delta\tilde{f}(\mathrm{k}_2) \right\rangle &= \sum_{\mathrm{r}_1,\mathrm{r}_2} d_r^{2D} e^{-\mathbb{I}\frac{\Delta\mathrm{k}\cdot\Delta\mathrm{r}}{2}} \bar{f} G_2(\Delta\mathrm{r}) e^{-2\mathbb{I}\bar{\mathrm{k}}\cdot\bar{\mathrm{r}}} \\
&= \int \mathrm{d}\mathrm{r}_1 \mathrm{d}\mathrm{r}_2 e^{-\mathbb{I}\frac{\Delta\mathrm{k}\cdot\Delta\mathrm{r}}{2}} \bar{f} G_2(\Delta\mathrm{r}) e^{-2\mathbb{I}\bar{\mathrm{k}}\cdot\bar{\mathrm{r}}} \\
&= \int \mathrm{d}\Delta\mathrm{r} e^{-\mathbb{I}\frac{\Delta\mathrm{k}\cdot\Delta\mathrm{r}}{2}} \bar{f} G_2(\Delta\mathrm{r}) \int \mathrm{d}\bar{\mathrm{r}} e^{-2\mathbb{I}\bar{\mathrm{k}}\cdot\bar{\mathrm{r}}}
\end{aligned} \tag{S23}$$

We explicitly write out the integral over $\bar{\mathrm{r}}$ for $D = 1$:

$$\begin{aligned}
\int \mathrm{d}\bar{\mathrm{r}} e^{-2\mathbb{I}\bar{\mathrm{k}}\cdot\bar{\mathrm{r}}} &= \int_{\frac{-L_r+|\Delta r|}{2}}^{\frac{L_r-|\Delta r|}{2}} \mathrm{d}\bar{\mathrm{r}} e^{-2\mathbb{I}\bar{\mathrm{k}}\bar{\mathrm{r}}} \\
&= \frac{\sin\left(\bar{k}(L_r - |\Delta\mathrm{r}|)\right)}{\bar{k}}
\end{aligned} \tag{S24}$$

If we take L_r to be much larger than any characteristic length scale for $G_2(\Delta\mathrm{r})$, namely, $G_2(\Delta\mathrm{r})$ is non-zero only for $|\Delta\mathrm{r}| << L_r$, the quantity $L_r - |\Delta\mathrm{r}|$ will be close to L_r and and thus very large. Accordingly, unless $\bar{k}$ is very close to zero, $\sin\left(\bar{k}(L_r - |\Delta\mathrm{r}|)\right)$ will become highly oscillatory and have a zero effect on average. Therefore, the above integral reduces to a delta function of $\bar{\mathrm{k}}$:

$$\int \mathrm{d}\bar{\mathrm{r}} e^{-2\mathbb{I}\bar{\mathrm{k}}\cdot\bar{\mathrm{r}}} \;\approx\; \pi\delta(\bar{\mathrm{k}}) \tag{S25}$$

which can be generalized to $\pi^D\delta(\bar{\mathrm{k}})$ in D dimensions.

Therefore, Eq. S23 becomes

$$\begin{aligned}\left\langle\delta\tilde{f}(\mathrm{k}_1)\delta\tilde{f}(\mathrm{k}_2)\right\rangle &\approx \pi^D\bar{f}\delta\left(\bar{\mathrm{k}}\right)\int \mathrm{d}\Delta\mathrm{r}e^{-\mathbb{I}\frac{\Delta\mathrm{k}}{2}\cdot\Delta\mathrm{r}}G_2(\Delta\mathrm{r}) \\ &= \pi^D\bar{f}\delta\left(\bar{\mathrm{k}}\right)\tilde{G}_2\left(\frac{\Delta\mathrm{k}}{2}\right), \end{aligned} \tag{S26}$$

where

$$\tilde{G}_2(\mathrm{p}) \equiv \int \mathrm{d}\mathrm{x}e^{-\mathbb{I}\mathrm{p}\cdot\mathrm{x}}G_2(\mathrm{x}) \tag{S27}$$

is the Fourier transform of the real-space equal-time two-point correlation function G_2, which is also the equal-time structure factor [51] (or under another name, the static structure factor [52]).

Equation S26 demonstrates a fundamental $\mathbf{r}-\mathbf{k}$ duality relation between the wave package that describes $\langle\delta f(\mathrm{r}_1)\delta f(\mathrm{r}_2)\rangle = \bar{f}G_2(\Delta\mathrm{r})$ and the wave package that decribes $\tilde{G}_2(\Delta\mathrm{k}/2)$(i.e. for $\left\langle\delta\tilde{f}(\mathrm{k}_1)\delta\tilde{f}(\mathrm{k}_2)\right\rangle$). If the former correlator has a large characteristic length scale in the real space, the latter correlator will have a small characteristic quasi-momentum scale with respect to $\Delta\mathrm{k}$. This duality relation corresponds to the following physical picture: when the system is strongly correlated with a diverging correlation length in the real space, the quasi-momentum cells that are associated with a quantum many-body system become decoupled to be nearly independent, leading to a simplified form of particle-hole symmetry breaking (as shown by Fig. 1a and Eq. 2 in the main text).

Under the above physical picture, we explore two examples regarding the Ising chain in a transverse field (see chapter 10 of Ref. [51]). In the first example, the equal-time two-point

correlation function of the order parameter at any finite temperature ($T > 0$) has an exponential decay form for large spatial separations [51]:

$$\lim_{|x|\to\infty} C(x,0) = Ae^{-|x|/\xi}, \tag{S28}$$

where ξ is the correlation length and A is a constant. Here $C(x,0)$ corresponds to a 1D version of the above $G_2(\Delta \mathrm{r})$ function. According to Eq. S26, the correlation function for the Fourier components in the quasi-momentum space (at small $|\Delta \mathrm{k}|$ values) is mainly determined by the Fourier transform of $C(x,0)$:

$$\tilde{C}\left(\frac{\Delta \mathrm{k}}{2},0\right) = \frac{4A}{\xi_k}\frac{1}{1+\frac{\Delta \mathrm{k}^2}{\xi_k^2}}, \tag{S29}$$

where

$$\xi_k = \frac{2}{\xi}. \tag{S30}$$

Equation S30 explicitly demonstrates the $\mathbf{r}-\mathbf{k}$ duality relation.

In the second example, right at zero temperature ($T = 0$) and at the quantum critical point, the equal-time correlator for the Ising chain system has a power-law dependence on the spatial separation $|x|$ [51]:

$$C(x,0) = \frac{A}{|x|^\alpha}, \tag{S31}$$

where the exponent α satisfies $0 < \alpha < 1$ (in the original example in Ref. [51], Section 10.4.3, α takes a value of 1/4; we here write a more general form for this exponent) and A is a coefficient. According to Eq. S26, the correlation function for the Fourier components in the

quasi-momentum space is mainly determined by $\tilde{C}$:

$$\tilde{C}\left(\frac{\Delta \mathrm{k}}{2},0\right) = \frac{\tilde{A}_\alpha}{|\Delta \mathrm{k}|^{1-\alpha}}, \tag{S32}$$

where $\tilde{A}_\alpha = 2^{2-\alpha}\sin(\frac{\alpha\pi}{2})\Gamma(1-\alpha)A$ is a coefficient that depends on α.

We see that for $\alpha = 1-\epsilon$ and $\epsilon \to 0^+$ (namely $C(x,0)$ decays relatively fast in the real space), the correlator $\tilde{C}$ decays slowly as $1/|\Delta \mathrm{k}|^\epsilon$. On the contrary, for $\alpha \to 0^+$, namely a "slowly-decaying" real-space equal-time two-point correlator $C(x,0)$, the correlator $\tilde{C}$ in quasi-momentum space decays relatively fast as $1/|\Delta \mathrm{k}|^1$.

In sum, the general derivations in this section, as well as the two examples based on Ref. [51], quantitatively demonstrate a fundamental $\mathbf{r}-\mathbf{k}$ duality relation. At a quantum critical point, the correlation length diverges as the temperature approaches zero [51,53]. Correspondingly, the $\mathbf{r}-\mathbf{k}$ duality relation suggests a certain type of vanishing characteristic correlation "length" scale in the quasi-momentum space, and thus *strongly motivates the decoupling of quasi-momentum cells into nearly independent cells, which leads to the simplified form of particle-hole symmetry breaking equation (Eq. 2 in the main text) that we expect to hold at and near a quantum critical point in arbitrary dimensions. Accordingly, non-mutual FES distribution [9,14] of quasi-particles (Eq. 3 in the main text) naturally emerges at and near a quantum critical point, as evidenced in the main text for one and two dimensions.*

4 Particle-hole symmetry breaking and the FES distribution

Here we show that the non-mutual FES distribution function can be directly obtained from particle-hole symmetry breaking that is a generic consequence of dynamical and statistical interactions [14] in arbitrary dimensions. For a quantum system, there are likely to exist such microscopic states for which the numbers of occupied states and of unoccupied states ($\rho(\mathbf{k})$ and $\rho_h(\mathbf{k})$) in a unit cell of phase space near quasi-momentum $\mathbf{k}$ are determined by the following form of particle-hole symmetry breaking

$$\rho_h(\mathbf{k}) + g\rho(\mathbf{k}) = d_{\mathrm{sp}}. \tag{S33}$$

Here g denotes a parameter that breaks down the particle-hole symmetry, and $d_{\mathrm{sp}} = 1/(2\pi)^D$ is the maximum dimensionality of the Hilbert space of available single-particle states in a phase-space unit cell for a non-interacting D-dimensional system. We will see that this parameter is nothing but the non-mutual FES parameter in Haldane's and Wu's formalism [8,9]. For free bosons, a state can be occupied by an infinite number of particles so that $\rho_h(\mathbf{k})$ is always equal to the density of all allowed states (d_{sp}). For free fermions, a state can be either occupied or unoccupied so $\rho(\mathbf{k}) + \rho_h(\mathbf{k}) = d_{\mathrm{sp}}$. However, for an interacting system such a symmetry between particles and holes is broken down; namely, either interaction or fractional exchange statistics can give rise to a form of Eq. S33. Here we consider the case of $0 \le g \le 1$.

We consider a cell of size $\mathrm{d}\mathbf{k}$ in the momentum space and the total number of states in

this cell is given by

$$\mathrm{d}W = \frac{[L(\rho(\mathbf{k}) + \rho_h(\mathbf{k}))\mathrm{d}\mathbf{k}]!}{[L\rho(\mathbf{k})\mathrm{d}\mathbf{k}]![L\rho_h(\mathbf{k})\mathrm{d}\mathbf{k}]!} \tag{S34}$$

The particle number N, energy E and entropy S are given by

$$N = L\int_{-\infty}^{\infty} \rho(\mathbf{k})\mathrm{d}\mathbf{k}, \quad E = L\int_{-\infty}^{\infty} \epsilon(\mathbf{k})\rho(\mathbf{k})\mathrm{d}\mathbf{k}, \quad S = L\int_{-\infty}^{\infty} \mathrm{d}W, \tag{S35}$$

respectively. Here $\epsilon(\mathbf{k})$ is the single particle energy. In the thermodynamic limit, $L\rho(\mathbf{k})\mathrm{d}\mathbf{k} \gg 1, L\rho_h(\mathbf{k})\mathrm{d}\mathbf{k} \gg 1$, the entropy can be written as

$$S \approx L\int_{-\infty}^{\infty} \mathrm{d}\mathbf{k}\left[(\rho(\mathbf{k}) + \rho_h(\mathbf{k}))\ln(\rho(\mathbf{k}) + \rho_h(\mathbf{k})) - \rho(\mathbf{k})\ln\rho(\mathbf{k}) - \rho_h(\mathbf{k})\ln\rho_h(\mathbf{k})\right]. \tag{S36}$$

The equilibrium states are determined by the minimization of the Gibbs free energy $G = E - TS - \mu N$, i.e., $\delta G = 0$ for any small changes of $\delta\rho(\mathbf{k})$ and $\delta\rho_h(\mathbf{k})$. From the particle-hole symmetry breaking equation, Eq. S33, we obatain

$$\delta\rho_h(\mathbf{k}) = -g\delta\rho(\mathbf{k}) \tag{S37}$$

and

$$\delta G = \delta E - T\delta S - \mu\delta N \tag{S38}$$

$$\approx L\int_{-\infty}^{\infty} \mathrm{d}\mathbf{k}\left[\epsilon - \mu - T(1-g)\ln(\rho + \rho_h) + T\ln\rho - Tg\ln\rho_h\right]\delta\rho \tag{S39}$$

Thus in the equilibrium, we have a dispersion relation

$$(1-g)\ln(\rho(\mathbf{k}) + \rho_h(\mathbf{k})) - \ln\rho(\mathbf{k}) + g\ln\rho_h(\mathbf{k}) = \frac{\epsilon(\mathbf{k}) - \mu}{T}. \tag{S40}$$

Let $w(\mathbf{k}) \equiv \rho_h(\mathbf{k})/\rho(\mathbf{k})$, the above equation becomes the well-known Haldane and Wu's FES equation [8,9,14]

$$(1 + w(\mathbf{k}))^{1-g} w(\mathbf{k})^g = e^{\frac{\epsilon(\mathbf{k})-\mu}{T}}. \tag{S41}$$

Furthermore, from the particle-hole symmetry breaking equation, Eq. S33, we obtain the FES distribution function

$$f(\mathbf{k}) = \frac{1}{w(\mathbf{k}) + g} \tag{S42}$$

where the number distribution function is given by $f(\mathbf{k}) = \rho(\mathbf{k})/d_{\rm sp}$. Similarly, we can further prove that the mutual FES can be directly obtained from the a general form of particle-hole symmetry breaking

$$\sum_{\mathbf{k}'} g(\mathbf{k}, \mathbf{k}')\rho(\mathbf{k}') + \rho_h(\mathbf{k}) = d_{\rm sp}. \tag{S43}$$

This result indicates that FES naturally comes from the particle-hole symmetry breaking relation (Eq. S33). Therefore FES is an emergent many-body phenomenon that depicts particle-hole symmetry breaking induced by either dynamical interaction or exchange statistics, enabling a quantum many-body system to be understood from the perspective of elementary excitations (depicted by non-interacting particles that obey FES). Consequently the FES description provides a powerful tool for understanding one- and higher dimensional many-body physics.

5 An ab initio computation: the emergence of simple, non-mutual FES for the low-energy excitations in 1D interacting gases

The Bethe ansatz equation (BAE) has the form [33]

$$\rho(k) + \rho_h(k) = \frac{1}{2\pi} + \int a(k-k')\rho(k')dk' \tag{S44}$$

where $\rho(k)$ and $\rho_h(k)$ are the particle and hole distribution functions respectively, and the kernel function $a(x) = \frac{1}{2\pi}\frac{2c}{c^2+x^2}$. In the strong coupling case $c/n \gg 1$, we can obtain

$$\begin{aligned}
\rho(k) + \rho_h(k) &= \frac{1}{2\pi} + \frac{1}{\pi c}\int \frac{1}{1+(k-k')^2/c^2}\rho(k')dk' \\
&\approx \frac{1}{2\pi} + \frac{1}{\pi c}\int \left(1 - \frac{(k-k')^2}{c^2}\right)\rho(k')dk' \\
&\approx \frac{1}{2\pi} + \frac{n}{\pi c} + O\left(\frac{1}{c^3}\right) \\
&\approx \frac{1}{2\pi}\left(1 + \frac{2n}{c}\right) + O\left(\frac{1}{c^3}\right)
\end{aligned}$$

Thus

$$\begin{aligned}
\frac{1}{2\pi} &\approx (\rho(k) + \rho_h(k))\left(1 + \frac{2n}{c}\right)^{-1} \\
&\approx (\rho(k) + \rho_h(k))\left(1 - \frac{2n}{c} + \frac{4n^2}{c^2}\right) \\
&= \rho(k)\left(1 - \frac{2n}{c} + \frac{4n^2}{c^2}\right) + \rho_h(k)\left(-\frac{2n}{c} + \frac{4n^2}{c^2}\right) + \rho_h(k) \\
&= \rho(k)\left[1 - \frac{2n}{c} + \frac{4n^2}{c^2} + e^{\frac{\varepsilon(k)}{T}}\left(-\frac{2n}{c} + \frac{4n^2}{c^2}\right)\right] + \rho_h(k)
\end{aligned} \tag{S45}$$

where $\varepsilon(k)$ is the dressed energy, i.e. $\rho_h(k)/\rho(k) = e^{\varepsilon(k)/T}$. Comparing the above result with the particle-hole symmetry breaking equation

$$g(k)\rho(k) + \rho_h(k) = \frac{1}{2\pi}, \tag{S46}$$

we can identify an explicit form for an effective non-mutual FES parameter $g(k) = \left(\frac{1}{2\pi} - \rho_{\mathrm{h}}(k)\right)/\rho(k)$ in the strong interacting case. In fact, there exists a region of quasi-momentum k such that

$$\frac{2n}{c}\left(1-\frac{2n}{c}\right)e^{\frac{\varepsilon(k)}{T}} \ll 1. \tag{S47}$$

Within this region, the non-mutual FES parameter only weakly depends on k:

$$g(k) = 1 - \frac{2n}{c}\left(1-\frac{2n}{c}\right)\left(1+e^{\frac{\varepsilon(k)}{T}}\right) \tag{S48}$$

Here, the density n and dressed energy $\varepsilon(k)$ in the strong initeracting case are given by [54]

$$n \approx -\frac{1}{2\sqrt{\pi}}T^{\frac{1}{2}}\mathrm{Li}_{\frac{1}{2}}\left(-e^{\frac{\mu}{T}}\right)\left[1-\frac{T^{\frac{1}{2}}}{\sqrt{\pi}c}\mathrm{Li}_{\frac{1}{2}}\left(-e^{\frac{\mu}{T}}\right)\right] + \frac{T}{2\pi c}\mathrm{Li}_{\frac{3}{2}}\left(-e^{\frac{\mu}{T}}\right)\mathrm{Li}_{-\frac{1}{2}}\left(-e^{\frac{\mu}{T}}\right) + O\left(\frac{1}{c^3}\right) \tag{S49}$$

$$\varepsilon(k) \approx k^2 - \mu + \frac{T^{3/2}}{\sqrt{\pi}c}\mathrm{Li}_{\frac{3}{2}}\left(-e^{\frac{\mu}{T}}\right)\left[1-\frac{T^{\frac{1}{2}}}{\sqrt{\pi}c}\mathrm{Li}_{\frac{1}{2}}\left(-e^{\frac{\mu}{T}}\right)\right] + O\left(\frac{1}{c^3}\right) \tag{S50}$$

Equations S48, S49, S50 provide an explicit analytic form of non-mutual FES physics that depicts the particle-hole symmetry breaking in the low-energy elementary excitations in 1D strongly interacting Bose gases.

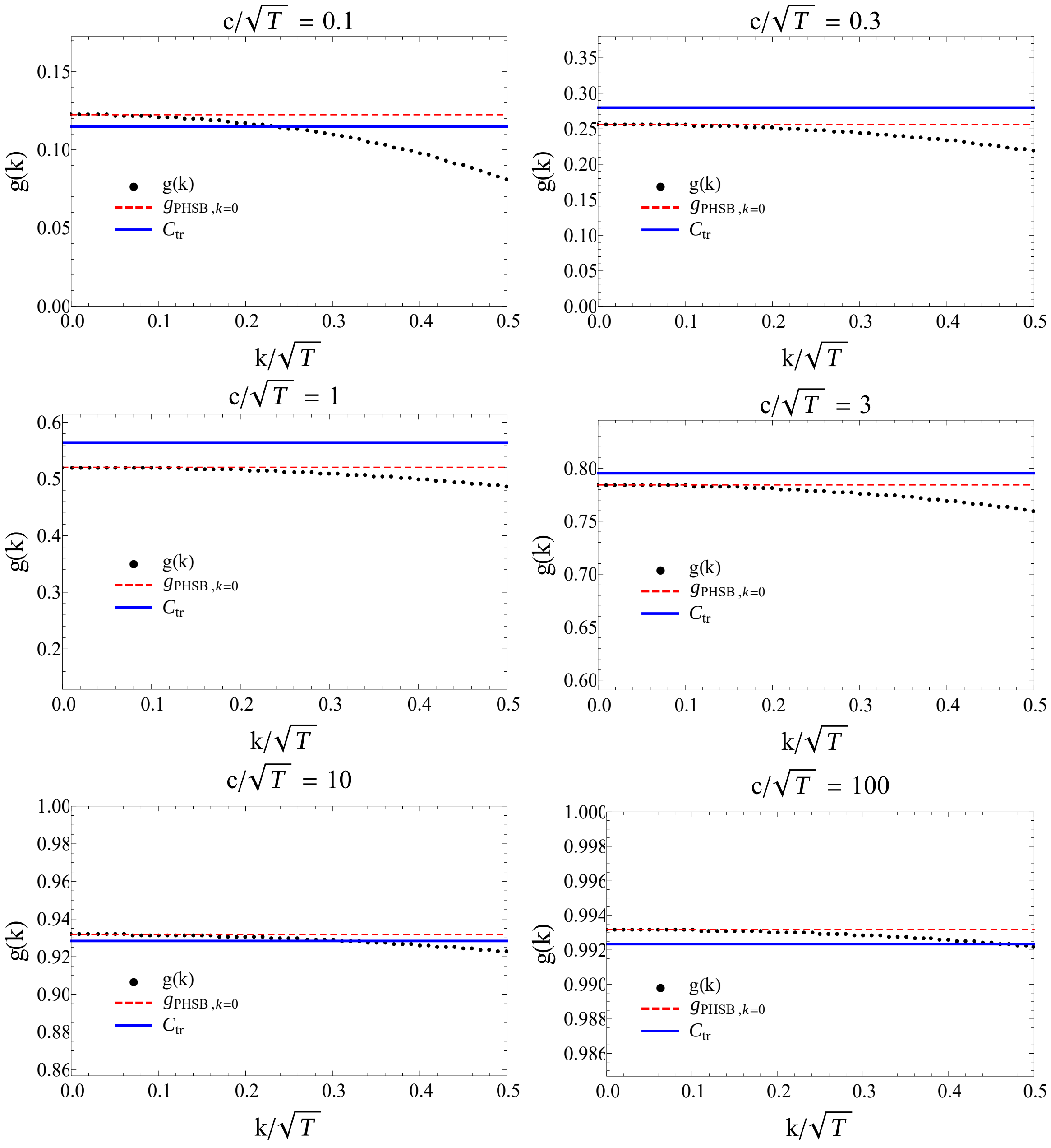

Figure S3: The effective non-mutual FES parameter $g(k)$ for 1D interacting gases at various interaction strengths. For $0.1 < \tilde{c} < \infty$, $g(k)$ is fairly homogeneous with respect to small k values within the range of $|k|/\sqrt{T} < 0.2$. For strongly interacting gases with large $\tilde{c}$, the homogeneity range becomes even wider.

While the above analytic form is available mostly in the strongly interacting regime, we use numerical computation to extend the concept of an emergent non-mutual FES physics for low-energy excitations in 1D interacting gases to an even wider range of interaction strengths. Here, at various scaled interaction strength $\tilde{c} = c/\sqrt{T}$, as long as the elementary excitations satisfy $|k| < O(\sqrt{T})$, the corresponding quasi-particles with characteristic energy well below the thermal energy scale share an almost homogeneous $g(k) \approx g_{_{\mathrm{PHSB},\mathbf{k}=0}}$, whose value depends on $\tilde{c}$ and is fairly close to $g_{\mathrm{max,1D}}\mathcal{C}_{\mathrm{tr}} = \mathcal{C}_{\mathrm{tr}}$, as shown in Fig. S3. The agreement between $g_{_{\mathrm{PHSB},\mathbf{k}=0}}$ and $\mathcal{C}_{\mathrm{tr}}$ shows that the latter indeed reveals the non-mutual FES nature of low-energy excitations. Based on the numerical results shown in Fig. S3, we also observe that practically, $g_{_{\mathrm{PHSB},\mathbf{k}=0}}$ is the maximum value of $g(k)$ and quantitatively depict the minimum extent to which the particle-hole symmetry has to be broken at a given interaction strength $\tilde{c}$.

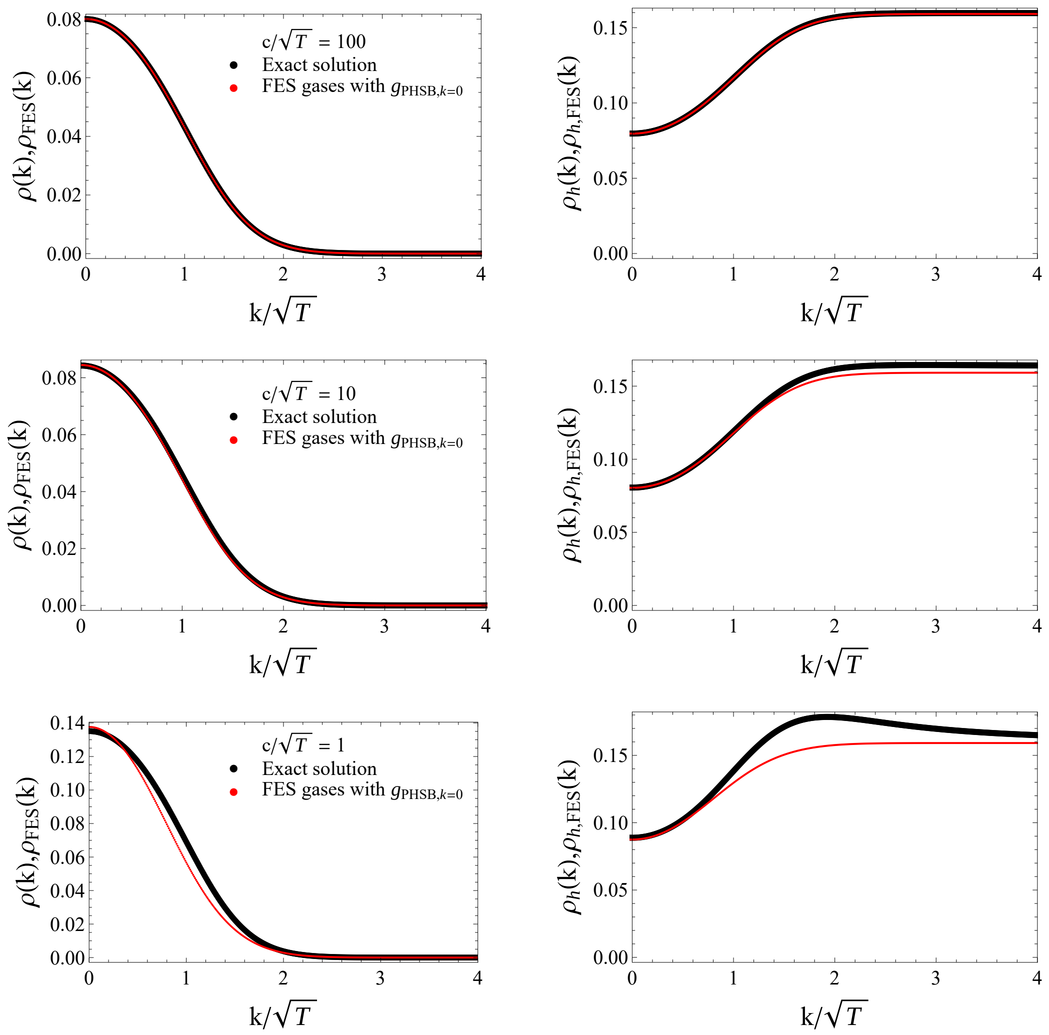

Figure S4: The non-mutual FES model (in red) associated with $g_{\mathrm{PHSB},\mathbf{k}=0}$ captures the essential behaviors of the exactly solved $\rho(k)$ and $\rho_{\mathrm{h}}(k)$ (in black) under various interaction strength $\tilde{c} = c/\sqrt{T}$.

Furthermore, as shown in Fig. S4, the non-mutual FES model associated with $g_{\mathrm{PHSB},\mathbf{k}=0}$ captures the essential behaviors of the exactly solved $\rho(k)$ and $\rho_{\mathrm{h}}(k)$ (in black) under various interaction strength $\tilde{c} = c/\sqrt{T}$. In particular, under strong interactions such as $\tilde{c} = 100$ and 10, $\rho_{\mathrm{FES}}(k)$ reproduces the exact solution very well. The overall good agreement between $\rho(k)$ and $\rho_{\mathrm{FES}}(k)$ and that between $\rho_{\mathrm{h}}(k)$ and $\rho_{\mathrm{h,FES}}(k)$ lays the foundation for the agreement between the thermodynamic observables of the 1D interacting Bose gases and those of the non-interacting gases of quasi-particles that obey FES.

6 Emergence of interaction-induced non-mutual FES in the strongly correlated regime at and near the quantum critical point

Besides Figs. 3 and 4 in the main text, we further benchmark the emergence of interaction-induced non-mutual FES in the strongly correlated regime at and near the quantum critical point. We explore the scope of application of Eq. 8 in the main text by comparing the scaled equations of state $\tilde{n}(\tilde{\mu}) \equiv \tilde{n}\left(\frac{\mu-\mu_{\mathrm{c}}}{T}\right)$ of interacting Bose gases with those of non-interacting FES quasi-particles in a finite range of $\tilde{\mu}$ besides the quantum critical point. With no additional parameters, a strongly interacting 1D Bose gas with $\tilde{c}_{\mathrm{1D}} = 100$ shows excellent equivalence to 1D non-interacting quasi-particles with $g = 0.992$ (Fig. S5a). As interaction weakens, equivalence at $\mu \leq \mu_{\mathrm{c}}$ is still good, whereas deviations become more significant as $\tilde{\mu}$ exceeds 0. Here we present $\tilde{n}$ because under the same $\tilde{c}$ and $\tilde{\mu}$, the agreement for $\tilde{p}$ and S/N is better than that for $\tilde{n}$ (see section 7). Fig. S5b shows similar scope of application of Eq. 8 (main text) in 2D, showing

equivalence between 2D interacting Bose gases and 2D non-interacting FES quasi-particles at and near the quantum critical point. In both 1D and 2D, interaction-induced non-mutual FES emerges and applies well in the strongly correlated regime at and near the critical point. Away from the critical point, we attribute the deviations at positive finite $\tilde{\mu}$ under weak interactions to mutual FES effects [14], see section 7 for details.

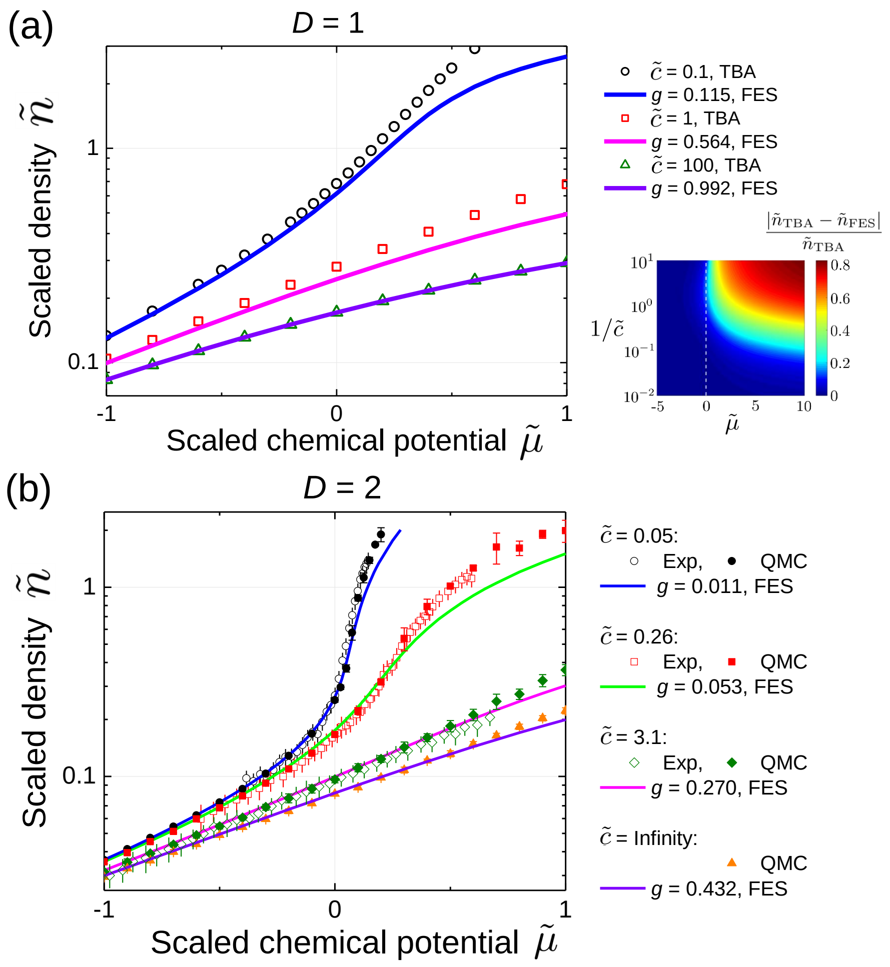

Figure S5: Emergence of interaction-induced non-mutual FES in the strongly correlated regime at and near the quantum critical point. (a) Scaled density $\tilde{n}$ as a function of $\tilde{\mu} = \frac{\mu - \mu_c}{T}$: 1D interacting Bose gases (open symbols) compared to 1D non-interacting particles with FES (lines). The colored map further illustrates the scope of application of non-mutual FES over various $\tilde{\mu}$ and $1/\tilde{c}$ values. (b) $\tilde{n}(\tilde{\mu})$ for 2D interacting gases: experimental measurements from Refs. 28, 32(open symbols) and QMC simulations (solid symbols), compared to $\tilde{n}(\tilde{\mu})$ for 2D non-interacting particles with FES (lines). The $\tilde{c}$-to-g mapping is based on Eq. 8 (main text) and independent of $\tilde{\mu}$.

7 Comparison between Yang-Yang equation and FES near the quantum critical point

While the mapping to non-mutual FES are obtained for critical point $\mu_c = 0$, we can further extend its scope of application to $\tilde{\mu} = \frac{\mu - \mu_c}{T} \neq 0$. To quantify the agreement between solutions to the Yang-Yang equation and the computation results based on FES, we define

$$\eta_\sigma \equiv \frac{|\sigma_Y - \sigma_F|}{\sigma_Y} \tag{S51}$$

where $\sigma = \tilde{n}, \tilde{p}, S/N$ and the subscripts "Y" and "F" denote the property obtained by Yang-Yang equation and FES, respectively. Our numerical results are shown in the Fig. S6. The contour plots of the deviations of density and pressure are respectively shown near the critical point, where 15%, 8% and 3% derivations are marked. In Fig. S5, we present $\tilde{n}$ because under the same $\tilde{c}$ and $\tilde{\mu}$, the relative deviations for $\tilde{p}$ and S/N are smaller than that for $\tilde{n}$.

The power of simple, non-mutual FES under strong interactions:

For a strong interaction, i.e. $\tilde{c} \gg 1$, these thermodynamic properties obtained from the interacting system and from the ideal particles with FES are in excellent agreement, even for large $\tilde{\mu}$. The bottom part of each panel in Fig. S6 illustrates this point.

The need of mutual FES under weak interactions:

On the other hand, under weak interactions, even when S/N shows fairly good agreement (Fig. S7, $\tilde{c} = 0.1$, $-1 \leq \tilde{\mu} < 0.5$), $\tilde{n}$ and $\tilde{p}$ show noticeable discrepancies in the same range, in particular for positive $\tilde{\mu}$ values, see Fig. S5a and Fig. S7, respectively. Thus these discrepancies are not caused by inaccuracy of our mapping formulae (Eqs. 8 and 9 in the main text) and cannot be alleviated by choosing a different "effective FES parameter g". Rather, such discrepancies in $\tilde{n}$ and $\tilde{p}$ are primarily due to the need of including more complex mutual FES effects [14]. We attribute these deviations at positive finite $\tilde{\mu}$, as well as the residual small deviations for $\tilde{\mu} \leq 0$, both primarily to the need for mutual FES effects [14], which is subject to future research.

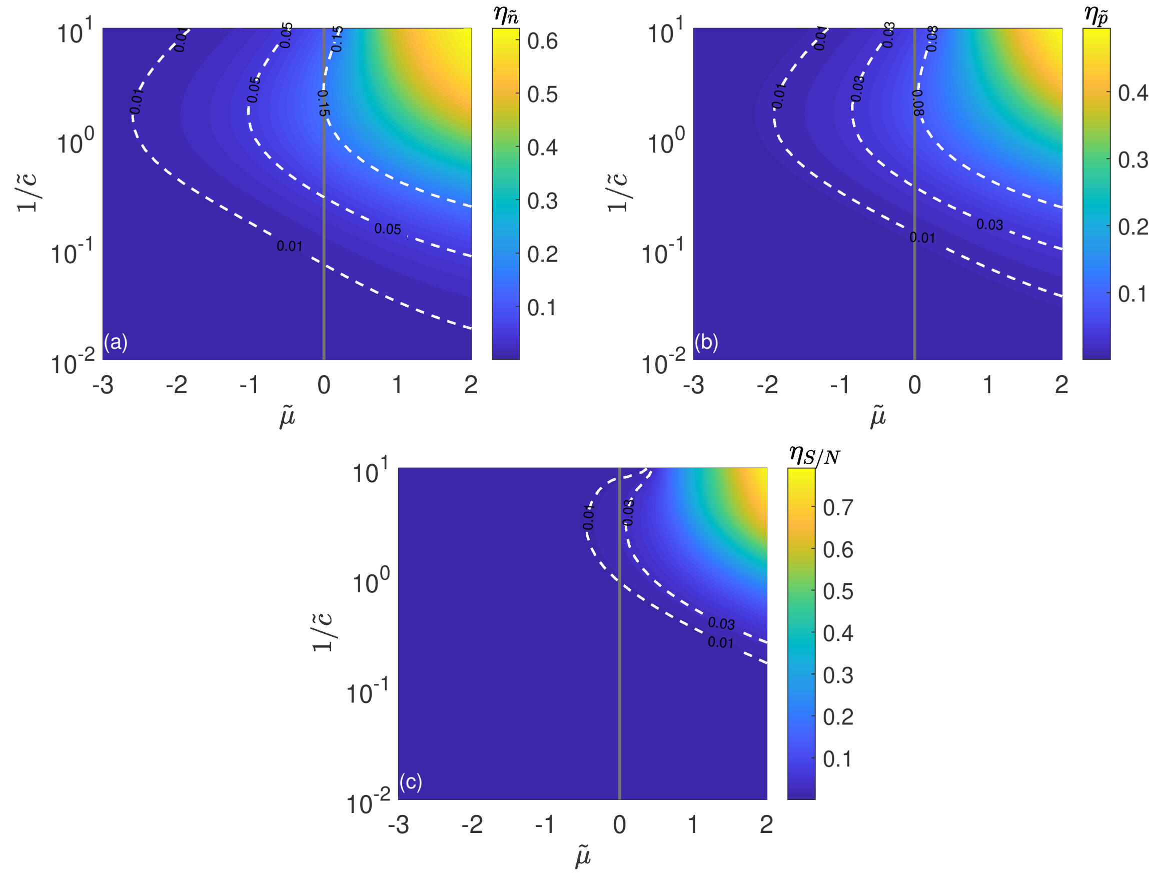

Figure S6: The comparison between interacting Bose gases and particles with non-mutual FES is shown in the chemical potential-interaction plane. **(a):** Contour plot of the density's deviation $\eta_{\tilde{n}}$. **(b):** Contour plot of the pressure's deviation $\eta_{\tilde{p}}$. **(c):** Contour plot of the entropy per particle's deviation $\eta_{S/N}$.

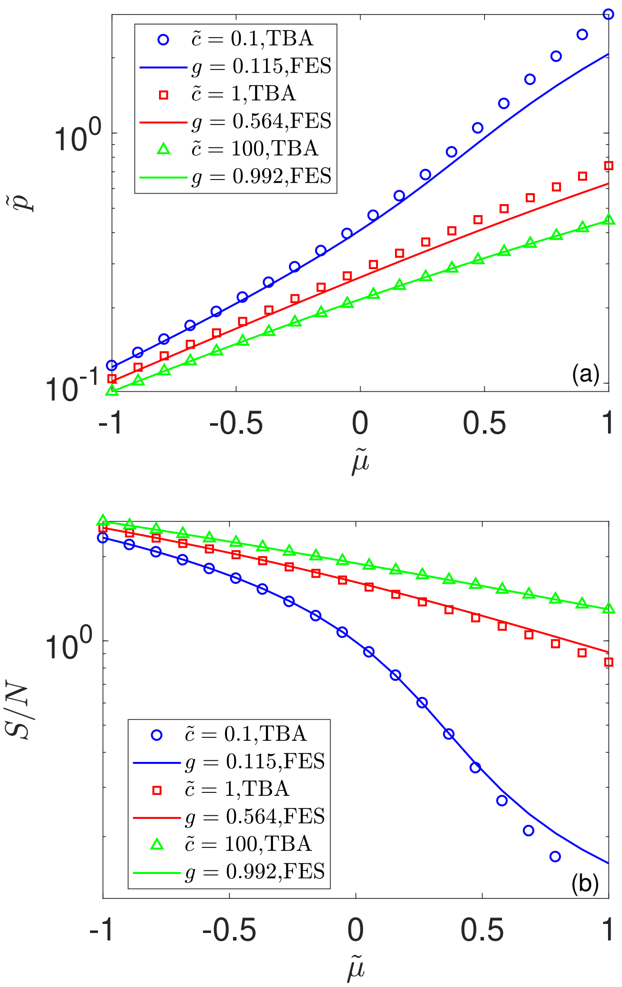

Figure S7: Scaled pressure $\tilde{p}$ and entropy per particle S/N as a function of scaled chemical potential $\tilde{\mu}$: 1D interacting Bose gases (open symbols) compared to 1D non-interacting particles with FES (lines).

8 Dimension analysis and dimensionless quantities

In this section and the following two sections, we will explicitly explain how we derive the dimensionless observables (particle density, pressure and entropy per particle, especially at the quantum critical point) in Bose gas in the continuous space based on the simulations for the Bose-Hubbard model on the lattice.

For D-dimensional ultracold Bose gas, the Hamiltonian could be written as [55,56]

$$\mathcal{H} = \sum_{i=1}^{N} \left(-\vec{\nabla}_i^2 - \mu \right) + c \sum_{i \neq j} \delta(\mathbf{r}_i - \mathbf{r}_j). \tag{S52}$$

This Hamiltonian has an equivalent field theory form

$$\mathcal{H} = \int d^D r \left\{ \hat{\psi}^\dagger(\mathbf{r}) \left(-\nabla^2 - \mu \right) \hat{\psi}(\mathbf{r}) + c \hat{\psi}^\dagger(\mathbf{r}) \hat{\psi}^\dagger(\mathbf{r}) \hat{\psi}(\mathbf{r}) \hat{\psi}(\mathbf{r}) \right\}, \tag{S53}$$

where $\hat{\psi}(\mathbf{r})$ is the wave function operator at the position $\mathbf{r}$.

By doing dimension analysis to Eq. S53, we obtain the dimension of the wavefunction operator and the parameters, that is

$$[\hat{\psi}(\mathbf{r})] = L^{-D/2}, \tag{S54}$$

$$[c] = EL^D, \tag{S55}$$

$$[\mu] = E, \tag{S56}$$

where E and L represent the dimension of energy and length, respectively.

If we choose E to be the unit of energy and λ to be the unit of length, we have the following relation equations between the quantities and their corresponding dimensionless quantities

$$\begin{aligned}
\mathbf{r} &= \lambda\tilde{\mathbf{r}} && \text{(S57)}\\
L &= \lambda\tilde{L} && \text{(S58)}\\
\nabla &= \lambda^{-1}\tilde{\nabla} && \text{(S59)}\\
\mu &= E\tilde{\mu} && \text{(S60)}\\
c &= E\lambda^{D}\tilde{c} && \text{(S61)}\\
\hat{\psi}(\mathbf{r}) &= \lambda^{-D/2}\tilde{\psi}(\tilde{\mathbf{r}}). && \text{(S62)}
\end{aligned}$$

Here, we add a tilde to each of the symbols to denote their dimensionless quantities; for convenience, the dimensionless quantity for $\hat{\psi}$ is denoted as $\tilde{\psi}$ with the hat dropped.

From Eq. S57, the Jacobian determinant is

$$Det\left(\partial\mathbf{r}/\partial\tilde{\mathbf{r}}\right) = \lambda^{D}. \tag{S63}$$

So, the integrals have the following relation equation

$$\int d^{D}rF(\mathbf{r}) = \left(\prod_{i=1}^{D}\int_{0}^{\tilde{L}} d\tilde{r}_i\right) Det\left(\partial\mathbf{r}/\partial\tilde{\mathbf{r}}\right)F(\lambda\tilde{\mathbf{r}}) = \int d^{D}\tilde{r}\lambda^{D}F(\lambda\tilde{\mathbf{r}}). \tag{S64}$$

Substituting these relation equations into Eq. S53, we can finally obtain the dimensionless form of the Hamiltonian

$$\begin{aligned}\tilde{\mathcal{H}}(\tilde{c},\tilde{\mu}) &= \mathcal{H}/E \\ &= \int d^D\tilde{r}\left\{\tilde{\psi}^\dagger(\tilde{\mathbf{r}})\left(-\frac{1}{E\lambda^2}\tilde{\nabla}^2-\tilde{\mu}\right)\tilde{\psi}(\tilde{\mathbf{r}})+\tilde{c}\tilde{\psi}^\dagger(\tilde{\mathbf{r}})\tilde{\psi}^\dagger(\tilde{\mathbf{r}})\tilde{\psi}(\tilde{\mathbf{r}})\tilde{\psi}(\tilde{\mathbf{r}})\right\}, \quad \text{(S65)}\end{aligned}$$

with the dimensionless parameters $\tilde{c} = c\lambda^{-D}E^{-1}$ and $\tilde{\mu} = \mu E^{-1}$.

Furthermore, if we take $E = T$ and $\lambda = \lambda_{dB}/(2\sqrt{\pi}) = 1/\sqrt{T}$, where $\lambda_{dB} = 2\sqrt{\pi/T}$ is the thermal de Broglie wavelength, we can get $E\lambda^2 = 1$, and the dimensionless Hamiltonian becomes

$$\begin{aligned}\tilde{\mathcal{H}}(\tilde{c},\tilde{\mu}) &= \mathcal{H}/T \\ &= \int d^D\tilde{r}\left\{\tilde{\psi}^\dagger(\tilde{\mathbf{r}})\left(-\tilde{\nabla}^2-\tilde{\mu}\right)\tilde{\psi}(\tilde{\mathbf{r}})+\tilde{c}\tilde{\psi}^\dagger(\tilde{\mathbf{r}})\tilde{\psi}^\dagger(\tilde{\mathbf{r}})\tilde{\psi}(\tilde{\mathbf{r}})\tilde{\psi}(\tilde{\mathbf{r}})\right\}, \quad \text{(S66)}\end{aligned}$$

with $\tilde{c} = cT^{D/2-1}$ and $\tilde{\mu} = \mu T^{-1}$. The corresponding dimensionless forms of those observables we are interested in, the particle number density, pressure and entropy per particle, become

$$\tilde{n} = n\lambda^D = nT^{-D/2}, \quad \text{(S67)}$$

$$\tilde{p} = p\lambda^D E^{-1} = pT^{-(D/2+1)}, \quad \text{(S68)}$$

$$\tilde{S}/\tilde{N} = S/N. \quad \text{(S69)}$$

As for the Bose-Hubbard model simulated in our numerical part of work, its Hamiltonian

reads

$$\mathcal{H}_{BH} = -t \sum_{<\vec{x},\vec{x}'>} \left(\hat{b}^\dagger_{\vec{x}}\hat{b}_{\vec{x}'} + \hat{b}^\dagger_{\vec{x}'}\hat{b}_{\vec{x}}\right) + \frac{U}{2}\sum_{\vec{x}} \hat{n}_{\vec{x}}(\hat{n}_{\vec{x}} - 1) - \mu_{BH}\sum_{\vec{x}} \hat{n}_{\vec{x}}, \tag{S70}$$

where $\vec{x}$ denotes the lattice site vector, $\hat{b}_{\vec{x}}$ ($\hat{b}^\dagger_{\vec{x}}$) is the annihilation (creation) operator for bosons on the site $\vec{x}$, $\hat{n}_{\vec{x}} = \hat{b}^\dagger_{\vec{x}}\hat{b}_{\vec{x}}$ is the particle number operator, and $< \vec{x}, \vec{x}' >$ indicates the summation runs over all the nearest neighbor sites. The parameter t is the tunneling parameter, U is the onsite interaction strength, and μ_{BH} is the chemical potential. Following the same approach, we can also obtain the dimensionless Hamiltonian for this model,

$$\tilde{\mathcal{H}}_{BH} = \frac{\mathcal{H}_{BH}}{T} = -\tilde{t} \sum_{<\vec{x},\vec{x}'>} \left(\tilde{b}^\dagger_{\vec{x}}\tilde{b}_{\vec{x}'} + \tilde{b}^\dagger_{\vec{x}'}\tilde{b}_{\vec{x}}\right) + \frac{\tilde{U}}{2}\sum_{\vec{x}} \tilde{n}_{\vec{x}}(\tilde{n}_{\vec{x}} - 1) - \tilde{\mu}_{BH}\sum_{\vec{x}} \tilde{n}_{\vec{x}}, \tag{S71}$$

where $\tilde{b}_{\vec{x}}$ is the dimensionless quantity for $\hat{b}_{\vec{x}}$, which is exactly $\hat{b}_{\vec{x}}$ itself since $\hat{b}_{\vec{x}}$ is already dimensionless, and $\tilde{t} = t/T$, $\tilde{U} = U/T$ and $\tilde{\mu}_{BH} = \mu_{BH}/T$ are the corresponding dimensionless quantities for each parameter, and the dimensionless forms of the observables are as follows

$$\tilde{n}_{BH} = n_{BH}, \tag{S72}$$

$$\tilde{p}_{BH} = p_{BH}/T, \tag{S73}$$

$$\tilde{S}_{BH}/\tilde{N}_{BH} = S_{BH}/N_{BH}. \tag{S74}$$

9 Mapping between Bose gases and the discrete Bose-Hubbard model

Since $\int d^D\tilde{r}\tilde{\psi}^\dagger(\tilde{\mathbf{r}})\tilde{\nabla}^2\tilde{\psi}(\tilde{\mathbf{r}}) = -\int d^D\tilde{r}\nabla\tilde{\psi}^\dagger(\tilde{\mathbf{r}}) \cdot \nabla\tilde{\psi}(\tilde{\mathbf{r}})$, the dimensionless form of the Hamiltonian for Bose gas, Eq. S66, can also be written as

$$\tilde{\mathcal{H}} = \int d^D\tilde{r}\left\{\tilde{\nabla}\tilde{\psi}^\dagger(\tilde{\mathbf{r}}) \cdot \tilde{\nabla}\tilde{\psi}(\tilde{\mathbf{r}}) + \tilde{c}\tilde{\psi}^\dagger(\tilde{\mathbf{r}})\tilde{\psi}^\dagger(\tilde{\mathbf{r}})\tilde{\psi}(\tilde{\mathbf{r}})\tilde{\psi}(\tilde{\mathbf{r}}) - \tilde{\mu}\tilde{\psi}^\dagger(\tilde{\mathbf{r}})\tilde{\psi}(\tilde{\mathbf{r}})\right\} \tag{S75}$$

In order to investigate the mapping relation between Bose gas and Bose-Hubbard model, we discretize the space into N^D small cells with the side length $\tilde{\Delta} = \tilde{L}/N$, where $\tilde{L}$ is the size of the system, making the integral in Eq. S75 into sums:

$$\tilde{\mathcal{H}} = \sum_{x_1=1}^{N} \sum_{x_2=1}^{N} \dots \sum_{x_D=1}^{N} \tilde{\Delta}^D \left\{ \tilde{\nabla} \tilde{\psi}_{\vec{x}}^{\dagger} \cdot \tilde{\nabla} \tilde{\psi}_{\vec{x}} + \tilde{c} \tilde{\psi}_{\vec{x}}^{\dagger} \tilde{\psi}_{\vec{x}}^{\dagger} \tilde{\psi}_{\vec{x}} \tilde{\psi}_{\vec{x}} - \tilde{\mu} \tilde{\psi}_{\vec{x}}^{\dagger} \tilde{\psi}_{\vec{x}} \right\}, \tag{S76}$$

where $\tilde{\psi}_{\vec{x}} \equiv \tilde{\psi}(\vec{x}\tilde{\Delta})$, and $\vec{x} = (x_1, x_2, ..., x_D)$ is the index of the cells with the integer components x_i ranging from 1 to N. $\tilde{\nabla}\tilde{\psi}_{\vec{x}}$ are numerical differences of $\tilde{\psi}_{\vec{x}}$ defined as

$$\tilde{\nabla} \tilde{\psi}_{\vec{x}} = \frac{1}{\tilde{\Delta}} \left(\tilde{\psi}_{\vec{x}+\vec{e}_1} - \tilde{\psi}_{\vec{x}}, \tilde{\psi}_{\vec{x}+\vec{e}_2} - \tilde{\psi}_{\vec{x}}, ..., \tilde{\psi}_{\vec{x}+\vec{e}_D} - \tilde{\psi}_{\vec{x}} \right). \tag{S77}$$

Thus, the discretized Hamiltonian becomes

$$\begin{aligned} \tilde{\mathcal{H}} &= -\tilde{\Delta}^{D-2} \sum_{<\vec{x},\vec{x}'>} \left(\tilde{\psi}_{\vec{x}}^{\dagger} \tilde{\psi}_{\vec{x}'} + \tilde{\psi}_{\vec{x}'}^{\dagger} \tilde{\psi}_{\vec{x}} \right) + \tilde{c} \tilde{\Delta}^D \sum_{\vec{x}} \tilde{\psi}_{\vec{x}}^{\dagger} \tilde{\psi}_{\vec{x}}^{\dagger} \tilde{\psi}_{\vec{x}} \tilde{\psi}_{\vec{x}} \\ &\quad - \left(\tilde{\mu} \tilde{\Delta}^D - 2D \tilde{\Delta}^{D-2} \right) \sum_{\vec{x}} \tilde{\psi}_{\vec{x}}^{\dagger} \tilde{\psi}_{\vec{x}} \end{aligned} \tag{S78}$$

If we make the replacement

$$\begin{aligned} \tilde{\psi}_{\vec{x}} &= \tilde{\Delta}^{-D/2} \tilde{b}_{\vec{x}}, \\ \tilde{\psi}_{\vec{x}}^{\dagger} &= \tilde{\Delta}^{-D/2} \tilde{b}_{\vec{x}}^{\dagger}, \end{aligned} \tag{S79}$$

we can get

$$\begin{aligned} \tilde{\mathcal{H}} &= -\tilde{\Delta}^{-2} \sum_{<\vec{x},\vec{x}'>} \left(\tilde{b}_{\vec{x}}^{\dagger} \tilde{b}_{\vec{x}'} + \tilde{b}_{\vec{x}'}^{\dagger} \tilde{b}_{\vec{x}} \right) + \tilde{c} \tilde{\Delta}^{-D} \sum_{\vec{x}} \tilde{n}_{\vec{x}} (\tilde{n}_{\vec{x}} - 1) \\ &\quad - \left(\tilde{\mu} - 2D \tilde{\Delta}^{-2} \right) \sum_{\vec{x}} \tilde{n}_{\vec{x}}. \end{aligned} \tag{S80}$$

By comparing Eqs. S80 and S71, we obtain the mapping relations between Bose gas in the continuous space and Bose-Hubbard model in the lattice as follows

$$\tilde{t} = \tilde{\Delta}^{-2}, \tag{S81}$$

$$\tilde{U} = 2\tilde{c}\tilde{\Delta}^{-D}, \tag{S82}$$

$$\tilde{\mu}_{BH} = \tilde{\mu} - 2D\tilde{\Delta}^{-2}, \tag{S83}$$

or reversely,

$$\tilde{\Delta} = \tilde{t}^{-1/2} = (T/t)^{1/2}, \tag{S84}$$

$$\tilde{c} = \frac{1}{2}\tilde{U}\tilde{\Delta}^{D} = \frac{1}{2}(U/t)(T/t)^{D/2-1}, \tag{S85}$$

$$\tilde{\mu} = \tilde{\mu}_{BH} + 2D\tilde{t} = (\mu_{BH}/t + 2D)(T/t)^{-1}. \tag{S86}$$

From the last equation, we can know that the critical point in Bose gas $\mu = 0$ corresponds to $\mu_{BH} = -2Dt$, that is, the critical point for the phase transition from a vacuum phase to a quantum liquid phase in Bose-Hubbard model.

With some more analysis, we can also obtain the mapping relationships between the observables in both models as follows

$$\tilde{n} = \tilde{n}_{BH}\tilde{\Delta}^{-D} = n_{BH}(T/t)^{-D/2}, \tag{S87}$$

$$\tilde{p} = \tilde{p}_{BH}\tilde{\Delta}^{-D} = (p_{BH}/t)(T/t)^{-(D/2+1)}, \tag{S88}$$

$$\tilde{S}/\tilde{N} = \tilde{S}_{BH}/\tilde{N}_{BH} = S_{BH}/N_{BH}. \tag{S89}$$

What we need to notice is that the corresponding dimensionless observables in Bose-Hubbard

model for $\tilde{n}$ and $\tilde{p}$ are not simply $\tilde{n}_{BH}$ and $\tilde{p}_{BH}$.

10 Extrapolation towards zero temperature

The extrapolation protocol and its application to 1D Bose-Hubbard systems According to the discretization approximation above, we would expect that when the dimensionless parameters $\tilde{c}$, $\tilde{\mu}$ in Bose gas model and ratios U/t, μ_{BH}/t, T/t in Bose-Hubbard model are associated by Eq. S85 and Eq. S86, the corresponding dimensionless observables in two systems are equivalent to each other except a correction brought by the finite dimensionless spacing $\tilde{\Delta}$, which could be expressed by the following equation

$$\tilde{O}(\tilde{g}, \tilde{\mu}) \quad = \quad \tilde{O}_{BH}(U/t, \mu_{BH}/t, T/t) + \tilde{f}(\tilde{g}, \tilde{\mu}, \tilde{\Delta}), \tag{S90}$$

where $\tilde{O}$ and $\tilde{O}_{BH}$ are two corresponding dimensionless observables in interacting Bose gas and Bose-Hubbard model respectively, and $\tilde{f}$ is the dimensionless correction function decaying to zero when $\tilde{\Delta}$ is approaching to zero, that is

$$\lim_{\tilde{\Delta} \to 0} \tilde{f}(\tilde{g}, \tilde{\mu}, \tilde{\Delta}) = 0. \tag{S91}$$

From Eq. S84, we know that the correction function could be rewritten as a function of the temperature, that is $\tilde{f}(\tilde{g}, \tilde{\mu}, T/t)$, and $\tilde{\Delta} \to 0$ is equivalent to $T/t \to 0$. Thus Eq. S90 and Eq. S91 becomes

$$\tilde{O}(\tilde{g}, \tilde{\mu}) = \tilde{O}_{BH}(U/t, \mu_{BH}/t, T/t) + \tilde{f}(\tilde{g}, \tilde{\mu}, T/t), \tag{S92}$$

and

$$\lim_{T/t\to 0} \tilde{f}(\tilde{g}, \tilde{\mu}, T/t) = 0. \tag{S93}$$

So, for the Bose-Hubbard model, only when $T/t \to 0$ will the dimensionless observables collapse to the corresponding dimensionless ones in continuous-space Bose gases.

Concluding from the analysis above, in order to obtain a dimensionless observable in Bose gas at $\tilde{c}$ and $\tilde{\mu}$, we can first compute the corresponding dimensionless observables in Bose-Hubbard model at different temperatures with the parameter ratios U/t and $\mu_{_{BH}}/t$ determined by Eq. S85 and Eq. S86, and then extrapolate the results towards zero temperature, which corresponds to the results in a Bose gas with no lattices.

As a typical example shown in Fig. S8, we measure and compute the dimensionless observables $\tilde{S}/\tilde{N}$ (equivalent with S/N) and $\tilde{n}$ at the critical point in one-dimensional Bose-Hubbard model by the formulas Eq. S87 and S89 with $\tilde{c} = 1$ and the temperature T/t varying from 1.0 to 0.1. We extrapolate these results to zero temperature by different formulas with different ranges of the temperature, and according to the distribution of these extrapolation results, we obtain the final estimates $S_c/N(\tilde{c} = 1) = 1.602(12)$ and $\tilde{n}_c(\tilde{c} = 1) = 0.28042(66)$, which are consistent with the theoretical results 1.602509 and 0.280377 obtained from solutions to the thermodynamic Bethe ansatz equation. Here, during the extrapolation, we apply rigorous statistics standards and only accept those fitting routines that can describe all data in the fitting ranges within 3 times of their error bars.

A discussion on scale invariance and the extrapolation towards zero temperature As shown by Eq. S6, the 1D interacting boson system studied in this work satisfies “scale invariance”, namely, numerical or experimental data taken at different temperatures can “collapse” onto universal functions (S/N versus $\tilde{\mu}$, $\tilde{n}$ versus $\tilde{\mu}$, $\tilde{p}$ versus $\tilde{\mu}$) when the parameters (quasi-momentum k, interaction strength c) and thermodynamic quantities are scaled properly according to the thermal de Broglie wavelength. On the other hand, a lattice gas system does not strictly satisfy scale invariance. To approach these universal functions using 1D Bose-Hubbard model, we need to reach a parameter regime where the dimensionless lattice spacing $\tilde{\Delta}$ is much smaller than all other relevant dimensionless length scales – and thus decoupled from the physical properties of the system. This is equivalent to requiring that the thermal de Broglie wavelength be much larger than all other relevant length scales. We further convert this requirement into a final criterion that the temperature be much lower than all other relevant energy scales – and thus decoupled from the physical properties of the system. Mathematically this is satisfied when T/t becomes sufficiently small. This is the physical motivation of our protocol of “extrapolation towards zero temperature”. In a practical simulation, all our numerical data are obtained under finite temperatures. So our extrapolation results represent the physical properties when the system temperature is much lower than all other relevant energy scales. As long as the temperature does not play a role in influencing the physical properties of the system, we consider the purpose of the extrapolation protocol to be fulfilled.

In 1D, we know beforehand that the system satisfies scale invariance, such that the ex-

trapolation towards zero temperature must have a well-defined limit. We indeed observe convergence in extrapolation (see Fig. S8), and observe that the QMC results (after extrapolation) agree excellently with the solutions to TBA equations (main text, Fig. 3). The statistical uncertainties of the extrapolated results reflect the accuracy of our QMC simulations and our confidence in using the QMC data to reveal the known physical and scaling properties of 1D Bose gases in continuous space. This computation serves as a calibration of our extrapolation protocol. When we apply this extrapolation protocol to 2D Bose-Hubbard lattice gases, where the corresponding continuous-space 2D Bose gases don't have an explicit equation like the TBA equation, *we do not presume a prerequisite that the continuous-space system satisfies scale invariance. Instead, we rely on the statistical uncertainties of the extrapolated results to provide understanding on the scaling behaviors and physical properties of the continuous-space Bose gases under sufficiently low temperatures.*

In an example shown in Fig. S9, we study 2D Bose-Hubbard model at $\tilde{c}_{2\mathrm{D}} = U/(2t) = 3$ and determine S_c/N, $\tilde{n}_c$, $\tilde{p}_c$ with the temperature T/t varying from 1.0 to 0.1. We extrapolate these results to zero temperature and obtain $S_c/N = 1.812(27)$, $\tilde{n}_c = 0.09787(94)$, and $\tilde{p}_c = 0.0876(16)$. We observe that the statistical uncertainties of the extrapolated results are fairly small, primarily because each individual numerical data point is accurately determined and has a small error bar (within $1 \sim 3\%$ level). While these results can potentially be further improved by future simulations with even lower simulation temperatures (T/t being on the 10^{-2} to 10^{-3} level), the current extrapolation results are sufficient to reveal the physical properties (S_c/N, $\tilde{n}_c$,

$\tilde{p}_c$) and scaling behaviors of the corresponding continuous-space Bose gases under sufficiently low temperatures.

As stated and shown in the main text (Fig. 4) and here (Fig. S5b), we obtain numerical results for 2D interacting Bose gases using QMC simulation data and the above extrapolation protocol. Our results agree with a non-perturbative renormalization group (NPRG) computation [46] for $\tilde{c}_{2\mathrm{D}} < 1$, and agree with experiments on 2D Bose gases without or with optical lattices [28,30–32] for $0.05 \leq \tilde{c}_{2\mathrm{D}} \leq 4.2$. The above-mentioned agreement supports our estimate on the physical properties of 2D Bose gases based on extrapolation. Hence our results for $\tilde{c}_{2\mathrm{D}} = 100, 200, 1000$, and ∞, obtained using the same extrapolation method and with similarly small statistical uncertainties, further provide new insights into a system of continuous-space 2D Bose gases with strong repulsive interactions (and with no inelastic losses in the model) under sufficiently low temperatures.

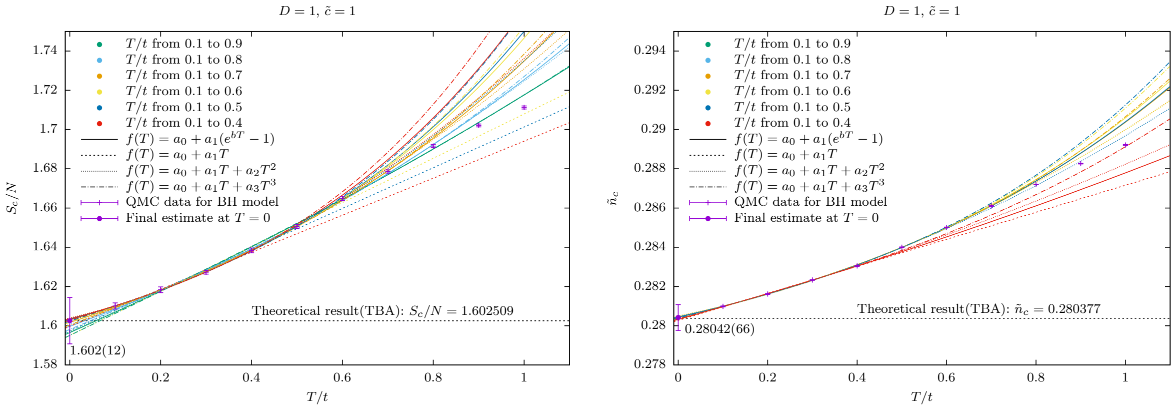

Figure S8: The entropy per particle and dimensionless particle number density at the quantum critical point, S_c/N and $\tilde{n}_c$, vs. T/t at $\tilde{c} = 1$ for one-dimensional Bose-Hubbard model. We apply different fitting formulas and fitting ranges of the temperature T/t shown in the figure to extrapolate the results towards zero temperature. Different colors of the lines indicate different fitting ranges, while different dash types of the lines represent different fitting formulas. Besides the formulas shown in this figure, some higher order of polynomials are also applied to do the extrapolation. According to the distribution of these extrapolation results, we obtain the final estimates as $S_c/N(\tilde{c} = 1) = 1.602(12)$ and $\tilde{n}_c(\tilde{c} = 1) = 0.28042(66)$, which are consistent with the theoretical results 1.602509 and 0.280377 derived by solutions to the thermodynamic Bethe ansatz (TBA) equations.

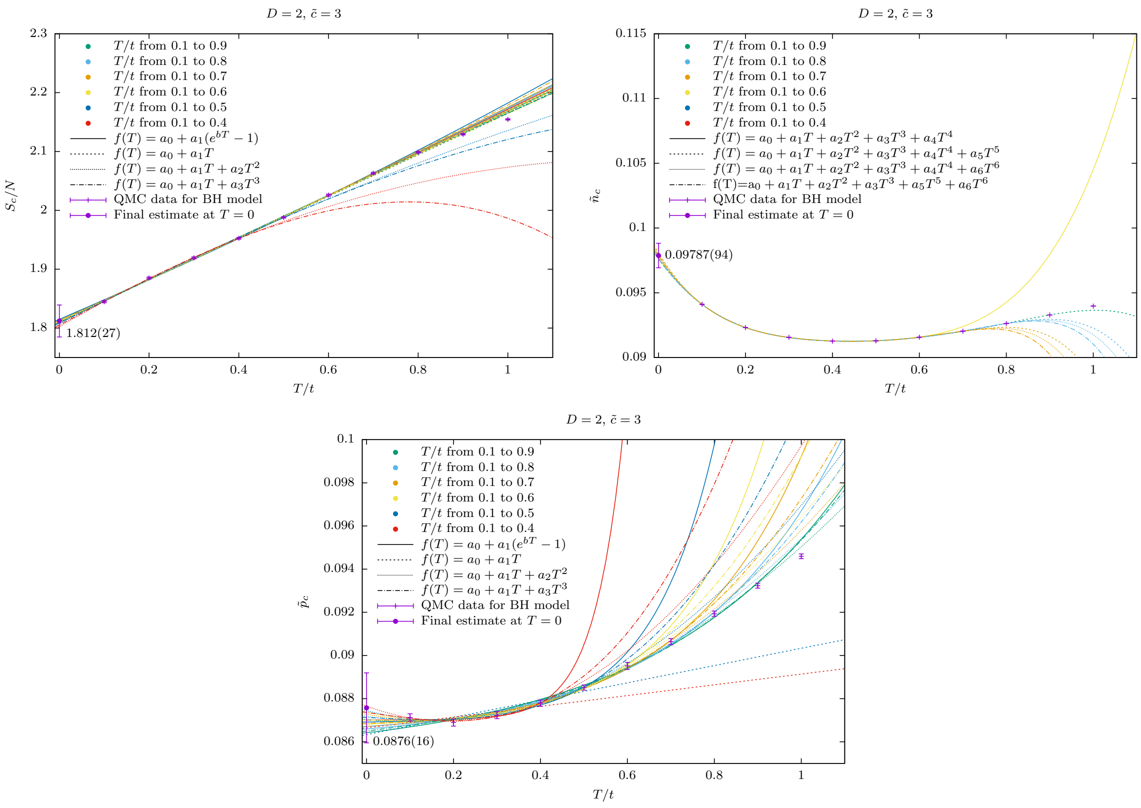

Figure S9: The entropy per particle, dimensionless scaled particle number density, and scaled pressure at the quantum critical point: S_c/N, $\tilde{n}_c$, $\tilde{p}_c$ vs. T/t at $\tilde{c}_{2\mathrm{D}} = 3$ for two-dimensional Bose-Hubbard model. We apply different fitting formulas and fitting ranges of the temperature T/t shown in the figure to extrapolate the results towards zero temperature. Different colors of the lines indicate different fitting ranges, while different dash types of the lines represent different fitting formulas.

11 Measuring of the observables in Bose-Hubbard model

In our work, we mainly focus on the following observables: particle density n, pressure p and entropy per particle S/N. In this section, we will show how we derive these observables in Bose-Hubbard model.

For both one-dimensional and two-dimensional systems, we apply worm algorithm in path-integral representation to simulate the Bose-Hubbard model by quantum Monte Carlo (QMC) method [44,45]. During the simulations, we can directly measure the particle number density $n_{_{BH}}$ and the grand energy density $\epsilon_{_{BH}} \equiv \frac{1}{V}\langle\mathcal{H}_{_{BH}}\rangle$, where V is the volume of the system, and in lattice model is just the total number of the sites. In order to ensure the data we obtained are in the thermodynamic limit, we keep the size of the system we simulated to be at least $L = 40t/T$, except when $T/t = 0.1$ we choose $L = 20t/T$ for some of them. A typical example in two dimension is shown in Fig. S10, and it presents that the system sizes we choose are large enough to allow us to neglect the errors brought by finite system sizes.

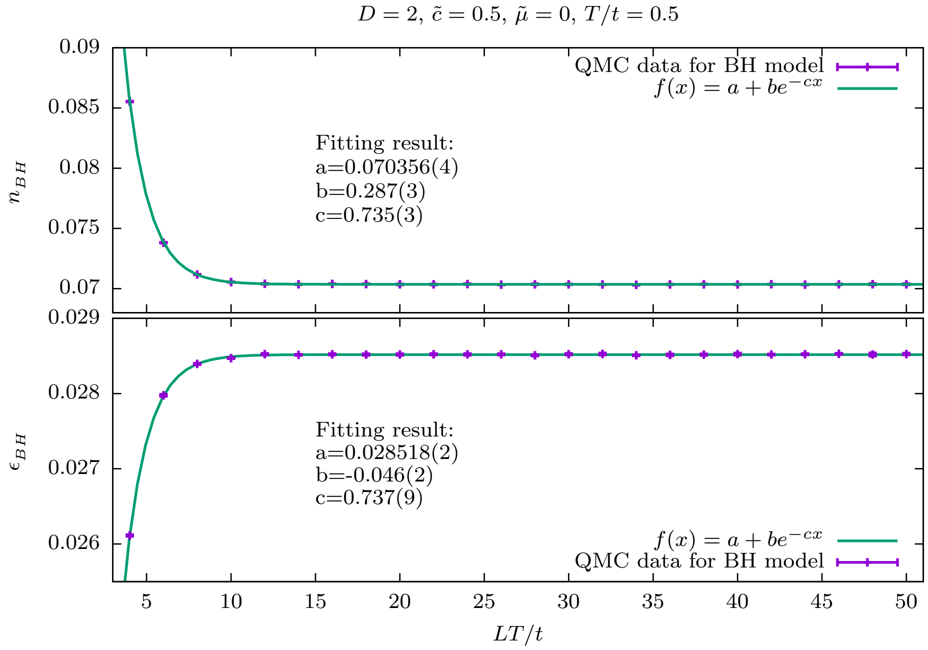

Figure S10: The particle number density and the grand energy density in Bose-Hubbard model, n_{BH} and ϵ_{BH}, vary with LT/t. Both curves could be well fitted by the formula $f(x) = a+be^{-cx}$, and according to the fitting results, even when $LT/t = 20$, the relative errors brought by the finite size of the system are of the order 10^{-6}, which is pretty small compared with the statistical relative errors (typically $10^{-4} \sim 10^{-3}$).

Since the particle number density for Bose-Hubbard model $n_{_{BH}}$ could be measured directly in our simulations, we will principally introduce how we derive the pressure $p_{_{BH}}$ and the entropy $S_{_{BH}}$ for Bose-Hubbard model.

According to the Gibbs-Duhem equation,

$$dp = nd\mu + sdT, \tag{S94}$$

if the temperature is fixed, we have

$$p(\mu) = p(\mu_0) + \int_{\mu_0}^{\mu} n(\mu')d\mu'. \tag{S95}$$

Considering that when $\mu \to -\infty$, the system will become vacuum with $n(\mu \to -\infty) = 0$ and $p(\mu \to -\infty) = 0$, we set $\mu_0 = -\infty$ and Eq. S95 becomes

$$p(\mu) = \int_{-\infty}^{\mu} n(\mu')d\mu', \tag{S96}$$

that is

$$p_{_{BH}}(\mu_{_{BH}}) = \int_{-\infty}^{\mu_{_{BH}}} n_{_{BH}}(\mu')d\mu' \tag{S97}$$

for Bose-Hubbard model. During our calculation of this formula, we break the integral into two parts: one is integrating from a very small value, say $\mu_{_{BH}}^{min}$, to $\mu_{_{BH}}$, while the other is integrating in the region below $\mu_{_{BH}}^{min}$, that is $(-\infty, \mu_{_{BH}}^{min})$. Therefore, we first measure $n_{_{BH}}$ at different chemical potentials ranging from $\mu_{_{BH}}^{min}$ to $\mu_{_{BH}}$, with other parameters U, t and T keeping fixed, and then calculate the integral in Eq. S97 numerically by trapezoidal rule in the

region $[\mu_{_{BH}}^{min}, \mu_{_{BH}}]$. And as for the region below $\mu_{_{BH}}^{min}$, we apply the distribution function for ideal Bosons

$$n(\varepsilon, \mu, T) = \frac{1}{e^{(\varepsilon-\mu)/T} - 1}, \tag{S98}$$

to fit the tail of our data for $n_{_{BH}}$ with the only fitting parameter ε, and based on the fitting result, we estimate the integral in the region $(-\infty, \mu_{_{BH}}^{min}]$. An example is shown in Fig. S11.

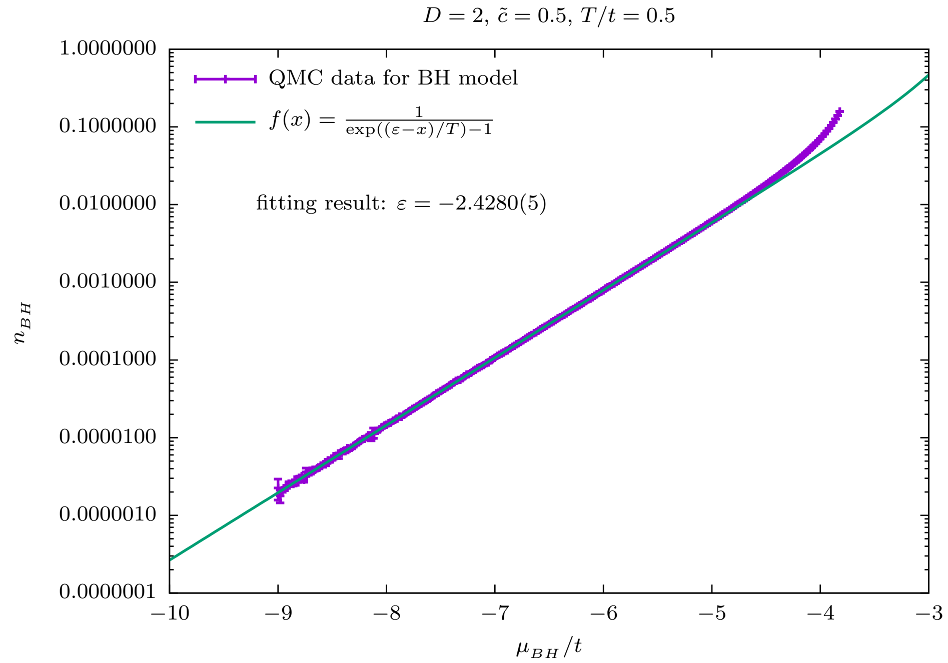

Figure S11: The particle number density in Bose-Hubbard model n_{BH} varies with the chemical potential μ_{BH}/t at the specific parameters shown in the title of the figure. In order to calculate the pressure at, for example, $\mu_{BH}/t = -4$, we measure n_{BH} in the region $\mu_{BH}/t \in [-9, -4]$ with the interval $\Delta\mu_{BH}/t = 0.02$, and calculate the integral in Eq. S97 numerically by trapezoidal rule and get $0.02686(6)$. We also fit the tail (here, we select $\mu_{BH}/t \in [-9, -6.5]$) of the data by the formula Eq. S98 with the fitting result $\varepsilon = -2.4280(5)$, and according to this, we obtain the estimate for the integral in the region $(-\infty, -9]$, which is around 1×10^{-6}. Thus, in fact, the relative deviation induced by the truncation is of the order 10^{-5} which is pretty small compared with the relative statistical errors($\sim 10^{-3}$). We have taken account of the integral from $-\infty$ to μ_{BH}^{min} in our results, but since μ_{BH}^{min} we choose are small enough, we could safely ignore the effect brought by the truncation in principle.

To calculate the pressure at the critical point p^c_{BH}, we typically set $\mu^{min}_{BH} = \mu^c_{BH} - 10T$ and the interval for the numerical integral $\Delta\mu_{BH} = 0.04T$, where $\mu^c_{BH} = -2Dt$ is the location of the critical point for the phase transition from a vacuum to a quantum liquid in Bose-Hubbard model. This corresponds to $\tilde{\mu}$ ranging from -10 to 0 with the dimensionless interval $\Delta\tilde{\mu} = 0.04$. As the example shown in Fig. S12, the errors coming from the finite interval during the numerical integrating can be ignored compared with the statistical errors.

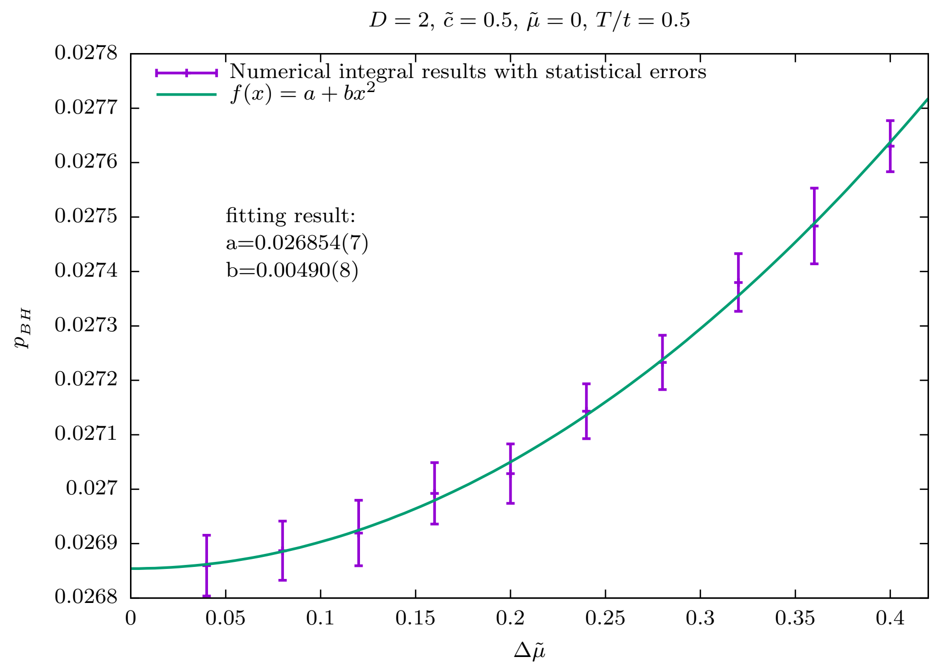

Figure S12: Numerical calculation results for the pressure in Bose-Hubbard model with different intervals $\Delta\tilde{\mu}$ during the numerical integrating. The error bars indicate the errors induced by the statistical uncertainty of the particle number density measured by QMC simulations. We can see that as the dimensionless interval $\Delta\tilde{\mu}$ decreases, the numerical integral results are approaching to a certain limit. This behavior could be well fitted by the formula $f(x) = a + bx^2$ as presented by the green line. According to the fitting result, the relative deviation resulting from the finite dimensionless interval is of the order 10^{-4} when $\Delta\tilde{\mu} = 0.04$, whereas the relative statistical errors are typically of the order 10^{-3}. Thus, under this circumstance, we could ignore the deviation brought by the finite interval during the numerical integrating.

In order to calculate the entropy of the system, we apply the following quasi-static process: keep all the parameters (including t, U and T) fixed except the chemical potential μ varying from μ_0 to μ extremely slowly, so that we could regard the system as always in equilibrium states. Following this process, we have

$$S(\mu) = S(\mu_0) + \int_{\mu_0}^{\mu} \left.\frac{\partial S}{\partial \mu}\right|_{\mu=\mu'} d\mu'. \tag{S99}$$

On the other hand, the entropy of a system is defined by the following formula

$$S = -Tr(\rho \ln \rho), \tag{S100}$$

where ρ is the density matrix with the explicit expression

$$\rho = e^{-\beta\mathcal{H}}/Z. \tag{S101}$$

Here, $Z = Tr\left(e^{-\beta\mathcal{H}}\right)$ is the partition function, $\mathcal{H}$ is the Hamiltonian in grand ensemble and typically could be written as $\mathcal{H} = \mathcal{H}_0 - \mu N$. According to Eq. S101, we could rewrite Eq. S100 in a more explicit form,

$$S = -\beta\frac{\partial \ln Z}{\partial \beta} + \ln Z. \tag{S102}$$

Therefore,

$$\frac{\partial S}{\partial \mu} = -\beta\frac{\partial}{\partial \mu}\frac{\partial \ln Z}{\partial \beta} + \frac{\partial \ln Z}{\partial \mu} = \beta\left(\frac{\partial}{\partial \mu}\langle\mathcal{H}\rangle + \langle N\rangle\right). \tag{S103}$$

Substitute this into Eq. S99 and we can get

$$S(\mu) \;=\; \int_{-\infty}^{\mu} \beta\left(\frac{\partial}{\partial \mu'}\langle\mathcal{H}(\mu')\rangle + \langle N(\mu')\rangle\right) d\mu'$$

$$
\begin{aligned}
&= \frac{1}{T}\left(\langle\mathcal{H}(\mu)\rangle + \int_{-\infty}^{\mu}\langle N(\mu')\rangle d\mu'\right) \\
&= \frac{V}{T}\left(\epsilon(\mu) + p(\mu)\right). \qquad \text{(S104)}
\end{aligned}
$$

For Bose-Hubbard model, it becomes

$$
S_{_{BH}}(\mu_{_{BH}}) = \frac{V}{T}\left[\epsilon_{_{BH}}(\mu_{_{BH}}) + p_{_{BH}}(\mu_{_{BH}})\right], \qquad \text{(S105)}
$$

where $\epsilon_{_{BH}}(\mu_{_{BH}})$ could be directly measured in our QMC simulations and $p_{_{BH}}(\mu_{_{BH}})$ could be obtained via the formula Eq. S97 by numerical integral techniques.

12 Comparing simulations with existing experimental measurements

First of all, We remark that our QMC data are obtained by spending about 2×10^5 CPU hours. It is surprising how well these data are described by the non-mutual FES model whose computation costs only tens of seconds. The agreement between theories, simulations, and experiments provides an excellent benchmark for interaction-induced particle-hole symmetry breaking (depicted by non-mutual FES) in 1D and 2D Bose gases.

For our theory and simulation results in both 1D and 2D systems, we compare them with existing experiments, as shown in Figs. 3 and 4 in the main text and Fig. S5. Below we briefly review the sources of experimental data and our re-analysis for some of them.

For the 1D gas experiment by the Kaiserslautern group, we directly obtain the $S_{\rm c}/N$ and $\tilde{n}_{\rm c}$ data from Ref. 29. For the 1D gas experiment by the USTC group [27], we obtain the original

data of equation of state ($n(\mu)$) that are taken under multiple temperatures, and re-analyze them to obtain the critical pressure p_c and the critical entropy density s_c based on $s = \left(\frac{\partial P}{\partial T}\right)_\mu$.

For the 2D gas experiment by the ENS, we obtain the S_c/N data directly from Ref. 30. For the 2D gas experiments by the Chicago group [28,32] where the 2D lattice gases / continuous-space gases are experimentally observed to satisfy scale invariance, we either obtain data from these two references or process the original data to extract the needed thermodynamic observables.

For another experiment by the Chicago group [31], we obtain the $\tilde{n}_c$ and $\tilde{p}_c$ data from Ref. 31, but do not have sufficient data to extract S_c/N because the authors neither test scale invariance in the strong coupling regime nor perform groups of measurements for the same $\tilde{c}_{2D}$ under multiple temperatures.

Finally, while our theoretical and numerical data agree quite well with existing experiments [27–32] in Figs. 3 and 4 of the main text and Fig. S5, we note that there are experimental factors that in principle can lead to the break-down of scale invariance to some extent in experimental systems – in particular, in the strongly interacting experimental systems. For example, three-body loss effects scale as the fourth power of the atomic scattering length. In addition, when one prepare strongly interacting experimental systems by approaching the unitarity limit (Feshbach resonance) or using deep optical lattices, the scattering amplitude at finite temperatures can become momentum-dependent. These and other experimental factors could in principle cause the break-down of scale invariance to some extent in experimental systems, but the

quantitative characterization of such break-down still remains sparse. The comparison of our theoretical and numerical data with existing experiments provide new insights regarding this issue. The overall good agreement suggests that in existing experiments, a description based on scale invariance is fairly consistent with the underlying physics within the current level of experimental uncertainties. The residual small discrepancies between our results and existing experiments may come from practical factors including inelastic losses and finite-temperature effects in experiments.

References for Supplementary Information

50. Jiang, Y.-Z., Chen, Y.-Y. & Guan, X.-W. Understanding many-body physics in one dimension from the lieb-liniger model. *Chinese Phys. B* **24**, 050311 (2015).

51. Sachdev, S. *Quantum Phase Transitions* (Cambridge University Press, 2011), second edn.

52. Hung, C.-L. *et al.* Extracting density–density correlations from in situ images of atomic quantum gases. *New Journal of Physics* **13**, 075019 (2011).

53. Continentino, M. A. Quantum critical point in heavy fermions. *Brazilian Journal of Physics* **35**, 197–203 (2005).

54. Guan, X.-W. & Batchelor, M. T. Polylogs, thermodynamics and scaling functions of one-dimensional quantum many-body systems. *J. Phys. A: Math. Theor.* **44**, 102001 (2011).

55. Greiner, M. Ultracold quantum gases in three-dimensional optical lattice potentials. *Ph.D. thesis* (2003).

56. Rey, A. M. Ultracold bosonic atoms in optical lattices. *Ph.D. thesis* (2004).
